# Mitotic catastrophe heterogeneity: implications for prognosis and immunotherapy in hepatocellular carcinoma

**DOI:** 10.3389/fimmu.2024.1409448

**Published:** 2024-07-01

**Authors:** Zun Mao, Zhixiang Gao, Ruyu Long, Huimin Guo, Long Chen, Sheng Huan, Guoping Yin

**Affiliations:** ^1^ Jiangsu Key Laboratory for Molecular and Medical Biotechnology, College of Life Sciences, Nanjing Normal University, Nanjing, China; ^2^ Department of Gastroenterology, The Affiliated Drum Tower Hospital of Nanjing University Medical School, Nanjing, China; ^3^ Department of Anesthesiology and Perioperative Medicine, The First Affiliated Hospital of Nanjing Medical University, Nanjing, China; ^4^ Department of Anesthesiology, Nanjing Second Hospital, Nanjing, China

**Keywords:** mitotic catastrophe, spatial transcriptome, prognosis, hepatocellular carcinoma, immune escape, machine learning

## Abstract

**Background and aims:**

The mitotic catastrophe (MC) pathway plays an important role in hepatocellular carcinoma (HCC) progression and tumor microenvironment (TME) regulation. However, the mechanisms linking MC heterogeneity to immune evasion and treatment response remain unclear.

**Methods:**

Based on 94 previously published highly correlated genes for MC, HCC patients’ data from the Cancer Genome Atlas (TCGA) and changes in immune signatures and prognostic stratification were studied. Time and spatial-specific differences for MCGs were assessed by single-cell RNA sequencing and spatial transcriptome (ST) analysis. Multiple external databases (GEO, ICGC) were employed to construct an MC-related riskscore model.

**Results:**

Identification of two MC-related subtypes in HCC patients from TCGA, with clear differences in immune signatures and prognostic risk stratification. Spatial mapping further associates low MC tumor regions with significant immune escape-related signaling. Nomogram combining MC riskscore and traditional indicators was validated great effect for early prediction of HCC patient outcomes.

**Conclusion:**

MC heterogeneity enables immune escape and therapy resistance in HCC. The MC gene signature serves as a reliable prognostic indicator for liver cancer. By revealing clear immune and spatial heterogeneity of HCC, our integrated approach provides contextual therapeutic strategies for optimal clinical decision-making.

## 1 Introduction

Primary liver cancer (PLC) is one of the most common malignant tumors globally and the leading cause of tumor-related deaths (1). Each year, over 700,000 new hepatocellular carcinoma (HCC) cases are diagnosed worldwide, with half occurring in China (2). PLC primarily includes HCC, intrahepatic cholangiocarcinoma, and mixed hepatocellular cholangiocarcinoma, with HCC being the most prevalent (3). HCC is challenging to diagnose early and progresses rapidly, resulting in poor prognosis. The American Joint Committee on Cancer (AJCC) classification is routinely used to assess HCC risk and treatment based on clinical stage. However, due to the complex tumor pathogenesis, individual differences, and tumor microenvironment (TME) heterogeneity, AJCC may be insufficient for accurately predicting prognosis and chemotherapy response. Furthermore, adjuvant chemotherapy (ACT) decisions based on clinicopathological staging, without considering molecular characteristics, risk inappropriate treatments (4). Therefore, exploring HCC molecular mechanisms and identifying new therapeutic targets is of significant scientific and clinical value.

Tumor cell proliferation, driven by excessive activation of mitosis-related signals, is a hallmark of cancer (1, 3). Mitotic catastrophe (MC) is a key regulatory mechanism in tumor cell death, triggered by issues such as spindle assembly defects and DNA damage, leading to cell division failure and programmed cell death via necrosis, apoptosis, senescence, or autophagy (5, 6). MC is characterized by abnormal cell division features like multinucleation, micronuclei, meganuclei, multipolar spindles, and polyploidy (7). MC disorders are involved in various liver diseases, including cirrhosis and liver tumors, playing a critical role in HCC progression, drug resistance, and immune evasion. Therefore, targeting MC in tumor cells offers a novel therapeutic perspective (8, 9).

Recent studies suggest that certain chemotherapeutic drugs may benefit HCC patients by activating the MC pathway. For instance, Taxol disrupts mitosis and induces cell death by stabilizing microtubules, forming multinucleated cells. A small molecule inhibitor, CGK733, enhances Taxol’s MC toxicity by targeting ATM and ATR kinase activity (10). Low-dose doxorubicin also induces HCC cell death through MC, forming cells with multiple micronuclei, while high-dose doxorubicin only induces apoptosis. Bcl-xL overexpression blocks apoptosis from high-dose doxorubicin but not MC and non-apoptotic death from low-dose doxorubicin (11, 12). Moreover, the HCC first-line treatment, sorafenib, can induce DNA replication errors and irregular mitosis, leading to MC and enhanced non-apoptotic liver injury by inhibiting Cyclins (E, A, and B) (13). Radiotherapy similarly causes MC through irreparable DNA double-strand breaks and micronuclei formation, leading to tumor cell mitotic failure (14).

The TME plays a crucial role in HCC development and recurrence, with potential interactions between MC and TME affecting tumor progression and immune response (15). Hypoxia in the TME may regulate MC either positively or negatively (16). Chronic biophysical constraints and AMPK-mediated molecular coevolution in the TME can promote chromosomal changes and mitosis progression in cancer cells (17). However, radiotherapy-induced MC may exacerbate TME conditions through micronuclei DNA fragmentation and senescence-associated secretory phenotype (SASP), necessitating senolytic drugs to clear senescent cancer cells (14). Targeted therapies may also influence tumor differentiation and metastasis by mediating the MC pathway (15).

This study utilized bioinformatics methods and predictive modeling to evaluate key MC regulatory genes in liver cancer progression and their correlation with TME. We aimed to identify immune associations with MC-related genes (MCGs) in HCC and explore the MC signature’s potential as a biomarker for HCC treatment.

## 2 Methods

The workflow of this study was shown in **Supplementary Figure S1**.

### 2.1 Publicly available cohort datasets and preprocessing

We obtained all MCGs from a previously published database (18), which currently contains 1,214 MC-associated genes and 5,014 compound data entries. From these, we selected 94 candidate genes with the highest feasibility level as our MCGs. We collected gene expression profiles of HCC and corresponding clinical datasets from the Gene Expression Omnibus (GEO) (19), The Cancer Genome Atlas (TCGA) (20), and the International Cancer Genome Consortium (ICGC) (21). We utilized gene expression and clinical information from GSE116174, GSE14520 (22), GSE45114 (23), and GSE76427 (24) for machine learning to screen the best prognostic prediction model. Additional datasets were used to evaluate our model’s efficiency: GSE104580 for transcatheter arterial chemoembolization (TACE) response, GSE223201 (25) for response to lenvatinib, GSE202069 (26) for anti-PD-L1 therapy response, GSE153203 (27) for combined lenvatinib and pembrolizumab treatment, GSE148355 (28) for HCC degree and tissue differences, GSE91061 (29) for melanoma anti-PD-L1/CTLA4 therapy, and the IMvigor210 cohort (30) for metastatic urothelial cancer anti-PD-L1 treatment.

### 2.2 Mutation and somatic copy number alteration analysis

We obtained mutation and SCNA data from GSCA: Gene Set Cancer Analysis (https://guolab.wchscu.cn/GSCA/#/) (31). In SCNA analysis, we classified a copy number of 2 as amplification and -2 as deep deletion. To minimize false positives, we retained only functional mutations (e.g., frameshift, nonsense, intragenic deletions/insertions). Using the maftools R package (32), we analyzed the tumor mutation annotation format (MAF) file and presented a gene mutation heatmap. Patient clinical and genomic data were also sourced from GSCA. Our survival analysis focused on the correlation between SCNA, mutation, and overall survival, while differential expression analysis compared tumor-related gene expression between SCNA and mutation positive/negative groups.

### 2.3 Consensus clustering analysis of MCGs

We identified HCC’s MC subtypes using consensus clustering analysis, applying an unsupervised algorithm with Euclidean distance and Ward linkage measures. The optimal cluster number was determined using the ConsensusClusterPlus R package (33), with 1,000 iterations to ensure stability. Principal component analysis then evaluated sample distribution differences between clusters. We compared the relationship between cluster-defined MC subtypes and clinicopathological characteristics (age, gender, stage, pathological type, grade) to evaluate clinical significance. The Kaplan-Meier method compared clustering’s impact on survival across datasets (p < 0.05 considered significant). We used the ggalluvial R package to create Sankey diagrams, visually displaying the correspondence between clustering and clinical variables. Finally, we presented gene expression patterns in different clusters via heatmaps.

### 2.4 Enrichment analysis: ssGSVA, GSEA, and WGCNA

Using the GSVA R package (34), we calculated functional annotation scores for gene sets of molecular subtypes and immune cell composition. Since most MCGs were negatively correlated with the MC process, we first calculated ssGSVA scores and then subtracted these scores from a constant to ensure a positive correlation with MC levels. Heatmaps were used to compare biological function differences between clusters. The Limma R package was used to screen differentially expressed genes (DEGs) between clusters. Based on DEGs and logFC values, we performed gene set enrichment analysis (GSEA) using the clusterProfiler R package and visualized the results. The c2.cp.kegg.v6.2 gene set from MsigDB was used for both GSVA and GSEA analyses (35).

Through Weighted Gene Co-expression Network Analysis (WGCNA) and dynamic tree cutting, we identified co-expression modules. We screened mRNA modules significantly related to MCG clusters and selected the highest correlation modules for further Gene Ontology (GO) and Kyoto Encyclopedia of Genes and Genomes (KEGG) enrichment analyses.

### 2.5 Functional hub gene analysis

We constructed the MCGs interaction network using the STRING database (36), setting the protein-protein interaction (PPI) score threshold to 0.7. After importing the network into Cytoscape software, we applied the EPC, MCC, Degree, and MNC algorithms in the cytoHubba plugin (37) to extract core network genes. Using the limma R package, we identified genes among the 94 MCGs with expression differences greater than 1.5-fold and adjusted p-values less than 0.05. By comparing these with core network genes, we identified key tumor-related genes. We analyzed differences in functional MCGs between normal and liver cancer tissues through the Human Protein Atlas (HPA). To verify clinical significance, we conducted pan-cancer expression and prognostic analysis of key genes on the GSCA platform using the TCGA database.

### 2.6 Machine learning for integrative construction of a consensus signature

To build a comprehensive risk scoring model with high accuracy and stability, we employed an integrated machine learning method (38) that combines 10 single models and 50 combination models for training. These models include Random Survival Forest (RSF), Supervised Principal Components (SuperPC), Least Absolute Shrinkage and Selection Operator (LASSO), and stepwise Cox regression. Our feature selection process was rigorous:

1. Single-factor Cox analysis screened MC related genes in the TCGA-LIHC datasets.
2. 50 algorithm combinations performed leave-one-out cross-validation to obtain a prediction model.
3. All models were tested on 6 independent validation datasets.
4. The Concordance index (C-index) of each model on the validation set was calculated.

### 2.7 Construction of the MC prognosis riskscore

We used LASSO and Cox regression models to screen excellent prognostic biomarkers from 94 candidate MCGs, determining the lambda value through 10-fold cross-validation (39). The LASSO Cox regression model was established using TCGA datasets as the training set, and multi-variable Cox analysis obtained regression coefficients for each gene to construct a Cox regression riskscore. The riskscore calculation formula is: Riskscore = ∑Coefficient of (gene i) × Expression of gene (i). We evaluated model accuracy using the C-index and Receiver Operating Characteristic (ROC) curve, calculating the Area Under the Curve (AUC) to compare with single biomarkers. Kaplan-Meier survival analysis verified the riskscore, and we drew 1, 3, and 5-year ROC curves to test model stability.

To further enhance prediction ability, we integrated T, N, M stage, overall stage, Child-Pugh level, and MC riskscore. Using the rms R package, we constructed survival nomograms for prognostic value prediction. This comprehensive approach ensures our model’s robustness and clinical applicability.

### 2.8 The role of MCGs signature in predicting immunotherapeutic and other therapeutic benefits

The IMvigor210 dataset includes 348 urothelial cancer cases with expression data, survival data, follow-up information, and immunotherapy response status. GSE91061 comprises 101 melanoma cases with gene expression, survival, follow-up, and immune data. GSE140901 contains comprehensive clinical data and immune efficacy for 24 HCC samples. Patients were categorized into PD, SD, PR, and CR groups based on their immune response. The MC riskscore was calculated using normalized count data to analyze its impact on prognosis and efficacy of PD-L1 inhibitors. GSE104580 was utilized to evaluate the riskscore’s predictive efficiency for TACE response. GSE223201 was obtained to assess riskscore differences following treatment with the VEGFR inhibitor lenvatinib in HCC. GSE202069 was used to evaluate the predictive efficiency of clinical response to anti-PD-L1 therapy in HCC patients. GSE153203 was employed to evaluate riskscore differences in HCC mice treated with a combination of lenvatinib and pembrolizumab.

### 2.9 Immune infiltration analysis

We quantified infiltration levels of 16 immune cell types and 13 immune-related pathways in each HCC sample using the ESTIMATE R package (40) on TCGA expression data. To validate the immune characteristics of MCGs clustering, we compared gene expression differences in major histocompatibility complexes and T cell stimulatory factors between clusters.

Using seven algorithms within the IORB R package (41)—TIMER, CIBERSORT, EPIC, QUANTISEQ, XCELL, and MCP-COUNTER—we determined differences in immune cell infiltration and function between high-risk and low-risk groups, displaying results graphically as a heatmap. We evaluated immune and stromal scores with the ESTIMATE algorithm and calculated differences in immune checkpoint genes between risk subgroups. Additionally, we predicted Immunophenoscore (IPS) for different riskscore subgroups.

To assess the relationships between clustering subtypes and immunotherapy effects, we employed immunotherapy response predictors: immune checkpoints, Tumor Immune Dysfunction and Exclusion (TIDE) score, and TME score.

### 2.10 Drug sensitivity analysis

We utilized IC50 values from two comprehensive databases for drug sensitivity analysis (42):

1. GDSC: 860 cell lines against 265 small molecule drugs.
2. CTRP: 1,001 cell lines against 481 small molecule drugs.

Using Pearson correlation analysis with FDR-adjusted p-values, we calculated the correlation between gene expression and drug IC50. Drugs were ranked based on their comprehensive correlation coefficient and FDR levels with our retrieved genes.

To evaluate binding energy and interaction modes between candidate drugs/small molecules and targets, we obtained:

1. Compound molecular structures from PubChem
2. Target protein structures from the Protein Data Bank (PDB)

We performed molecular docking studies using Autodock Vina 1.2.2 (43) for model visualization, ensuring a thorough and reproducible analysis of drug-target interactions.

### 2.11 Single-cell RNA sequencing analysis

We downloaded scRNA-seq data of 7 HBV-related HCC tissues from GSE202642 in GEO. Cells with less than 250 expressed genes were removed, and remaining genes were logarithmically normalized. We used ScaleData and SCTransform functions in Seurat R package to eliminate batch effects. For nonlinear dimensionality reduction, we employed uniform manifold approximation, selecting the first 13 of the 20 principal components.

Using FindNeighbors and FindClusters functions (dimensions = 20, resolution = 0.5), we clustered individual cells into subgroups. We performed UMAP dimensionality reduction with RunUMAP. We annotated cell types (cancer cells, endothelial cells, hepatocytes, fibroblasts, immune cells) through marker genes like EPCAM, MS4A1, CD79A, FGFBP2, CD68, ACTA2, PECAM1 (44).

We conducted scRNA trajectory analysis using monocle2 (45), intercellular communication analysis with italk (46), and PAGA analysis with SCP package (47).

### 2.12 Spatial transcriptome analysis

We obtained original chip data from GSM7661255 from a HCC patient who experienced recurrence after cabozantinib/nivolumab (CABO/NIVO) treatment from GEO (48). Data preprocessing used Seurat R package for background correction, normalization, and standardization (44).

Differential expression analysis with sub-cluster comparisons was performed using FindMarkers function. We annotated different tumor cell states from GSE202642 onto spatial data using Seurat’s FindTransferanchors algorithm. The Xcell scoring method defined relative gene-set enrichment scores of signal pathways in different regions (49).

Using COMMOT method, we inferred intercellular communication and spatial signal directionality specificity, along with key downstream genes, considering competition between different ligand-receptor (LR) pairs and spatial cell distances (50). For analyzing inter- and intra-cellular interactions, we utilized stLearning SCTP pipeline (51), which extracts significant spot/cell interactions, calculates LRscores based on co-expression information and cell type diversity, and maps results onto spatial distribution.

Applying multimodal intersection analysis (MIA) based on hypergeometric testing, we analyzed significant enrichment of specific cell gene-sets, identifying major cell types in various spatial regions (52).

We used SPATA2 for spatial trajectory analysis, exploring gene expression variations in tumor spatial heterogeneity (53). The runVectorFields function calculated vector field tables, indicating aggregated gene expression direction. The getSpatialTrajectoryIds function visualized spatial-specific changes from key MC genes, MC scores, and cell proliferation scores. For copy number variation (CNV) analysis, we aligned genes based on chromosome positions and assessed CNVs using moving averages with a 100-gene sliding window for each chromosome using InferCNV function.

To identify spot groups (NICHEs) with similar cell-type compositions across samples, we utilized isometric log ratios, transforming the estimated cell-type proportions of each spatial transcriptomics spot and slide (54). We employed mistyR package to assess the significance of major cell type abundance in explaining other major cell types’ abundance (54).

## 3 Results

### 3.1 Somatic mutation landscape of MCGs

**Figure 1:**
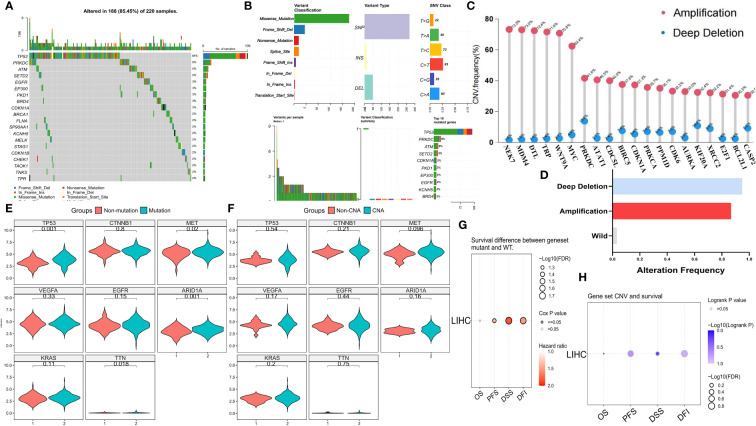
Genomic landscape of MCGs in HCC. **(A)** Landscape of genomic aberrations of the top 20 MCGs with mutation in HCC. **(B)** Summary of the mutation classes of MCGs in HCC. **(C)** Lollipop chart of the SCNA proportion in MCGs. **(D)** Histogram of the proportion of differential SCNA types in HCC. **(E, F)** Expression comparison of Tumor-related Genes between WT and MCGs mutation or SCNA in HCC. **(G)** Survival difference between MCGs WT and mutation. **(H)** Survival difference between MCGs WT and SCNA.

To investigate the genomic characteristics of MCGs in HCC, we visualized mutation and SCNA data for 369 HCC patients from the TCGA cohort (**Figures 1A–C**). Approximately 59.62% of patients had mutations in MCGs, with mutation frequencies ranging from 2% to 48% (**Figure 1A**). The MCGs with the highest mutation frequencies were TP53 (48%), PRKDC (9%), and ATM (5%), with predominant mutation types being missense, frameshift deletions, and nonsense mutations (**Figure 1B**). Amplification of NEK2, MDM4, DTL, TPR, and WNT9A was notably high (over 70.8%), with almost no deep deletions (**Figure 1C**). About 94.85% of HCC patients had at least one MCGs SCNA (**Figure 1D**). Most MCGs with high SCNA frequencies tended to be co-amplified rather than co-deleted. HCC patients with MCGs mutations had significantly higher expression levels of tumor-related genes, including MET, TP53, ARID1A, and TTN (**Figure 1E**), while no significant differences were found among patients divided by SCNA status (**Figure 1F**). The tumor mutation map displayed the distribution of tumor mutation burden (TMB) scores across various cancer types (**Supplementary Figure S2A**). Mutual exclusivity and co-occurrence analysis of the top 20 mutated MCGs revealed that TP53 mutations co-occurred with mutations in several MCGs (e.g., CDKN1A, PKD1, EGFR), with little mutual exclusivity observed (**Supplementary Figure S2B**). CDKN1A, TP53, and STAG1 had the highest variant allele frequencies (VAF) (**Supplementary Figure S2C**). Enrichment analysis showed significant enrichment of MCGs single nucleotide polymorphism (SNP) sites in cancer-related signaling pathways, including TP53, cell cycle, and RTK/RAS pathways (**Supplementary Figure S2D**). Immune infiltration analysis indicated higher gamma/delta T cell infiltration in the SCNA amplification group and higher CD4^+^ naive T cell infiltration in the SCNA deletion group (**Supplementary Figure S3A**). Compared to the high mutation group, the low mutation group exhibited greater immune cell infiltration (e.g., CD4^+^ naive, CD4^+^ T, Th2) (**Supplementary Figure S3B**). Progression-free survival (PFS), disease-free interval (DFI), and disease-specific survival (DSS) were significantly lower in the mutation group compared to the non-mutation group (**Figure 1G**), while no significant differences in overall survival (OS), PFS, DFI, or DSS were observed between SCNA and non-SCNA patients (**Figure 1H**). Kaplan-Meier curves for OS, PFS, DFI, and DSS are shown in **Supplementary Figures S3C** and **D**. Pan-cancer analysis also indicated minimal significant SCNA differences in prognosis across all tumors, while MCGs mutations were associated with significantly worse prognosis in LIHC, PAAD, PCPG, and other cancers (**Supplementary Figures S2E**, **S3E**). These findings suggest that mutations, rather than SCNAs, are the primary drivers of MCGs dysregulation in HCC patients, leading to poor prognosis.

### 3.2 Functional analysis of MCGs

Expression and pathway activity analysis demonstrated that apoptosis, cell cycle, DNA damage, and EMT pathways were significantly positively correlated with MCGs, whereas RAS/MAPK and PTK pathways were negatively correlated (**Supplementary Figure S4A**). Survival analysis indicated that patients with lower MC ssGSVA scores had significantly worse prognosis in OS, DSS, and PFS across most cancers (**Supplementary Figure S4B**). Significant ssGSVA score differences were also observed between normal and tumor tissues in pan-cancers, with higher scores in normal tissues (**Supplementary Figure S4C**). Strong correlation between ssGSVA score and enriched pathway showed significant concentration of MCGs gene functions (**Supplementary Figure S4D**). Correlation analysis between GSVA scores and immune pathways/cells showed a strong positive correlation between high MC levels and pathways such as IL and MHC/APC co-stimulation, and significant correlation with the infiltration of macrophages, Tfh, Th2, Treg, and other immune cells (**Supplementary Figure S4E**). GSEA enrichment analysis confirmed the enrichment of MCGs in HCC patients (**Supplementary Figure S4F**).

### 3.3 Identifying hub functional MCGs

**Figure 2:**
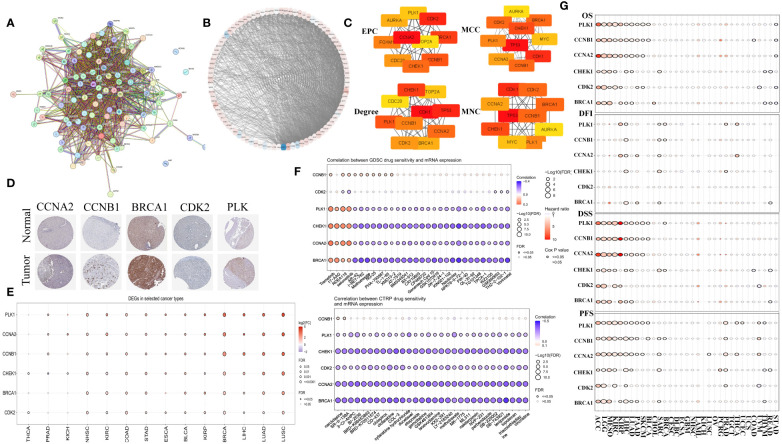
Identification of hub genes in MCGs protein-protein interaction network. **(A)** Identification of MCGs PPI network. **(B)** Circle layout visualization of the MCGs PPI. **(C)** Identification of 6 hub genes with EPC, MCC, Degree, MNC algorithm. **(D)** Comparison of protein expression of hub MGCs between normal and tumor tissue. **(E)** Comparison of mRNA expression of hub MGCs between normal and tumor in the Pan-cancer. **(F)** Drug sensitivity analysis of compound targeted MGCs in the GDSC and CTRP. **(G)** Summary of the survival difference of hub MCGs in the Pan-cancer.

The PPI network for MCGs was established using STRING (**Figures 2A, B**). By applying four built-in algorithms in Cytoscape (EPC, MCC, Degree, and MNC), we identified six hub genes: CCNA2, CCNB1, BRCA1, CDK2, PLK1, and CHEK1 (**Figure 2C**). Immunohistochemistry analysis revealed higher protein expression of these hub MCGs in tumor tissue (except for CHEK1) via the HPA website (**Figure 2D**). Pan-cancer mRNA comparison confirmed higher expression of these genes in tumor versus normal tissue (**Figure 2E**), and drug sensitivity analysis using GDSC and CTRP suggested compounds like AR-42, I-BET-762, 3-CI-AHPC, and BI-2536 as potential MC therapeutics (**Figure 2F**). Survival analysis demonstrated that almost all six hub MCGs were associated with poorer prognosis in liver cancer and other cancers (**Figure 2G**).

### 3.4 Identification of MC subtypes in HCC

**Figure 3:**
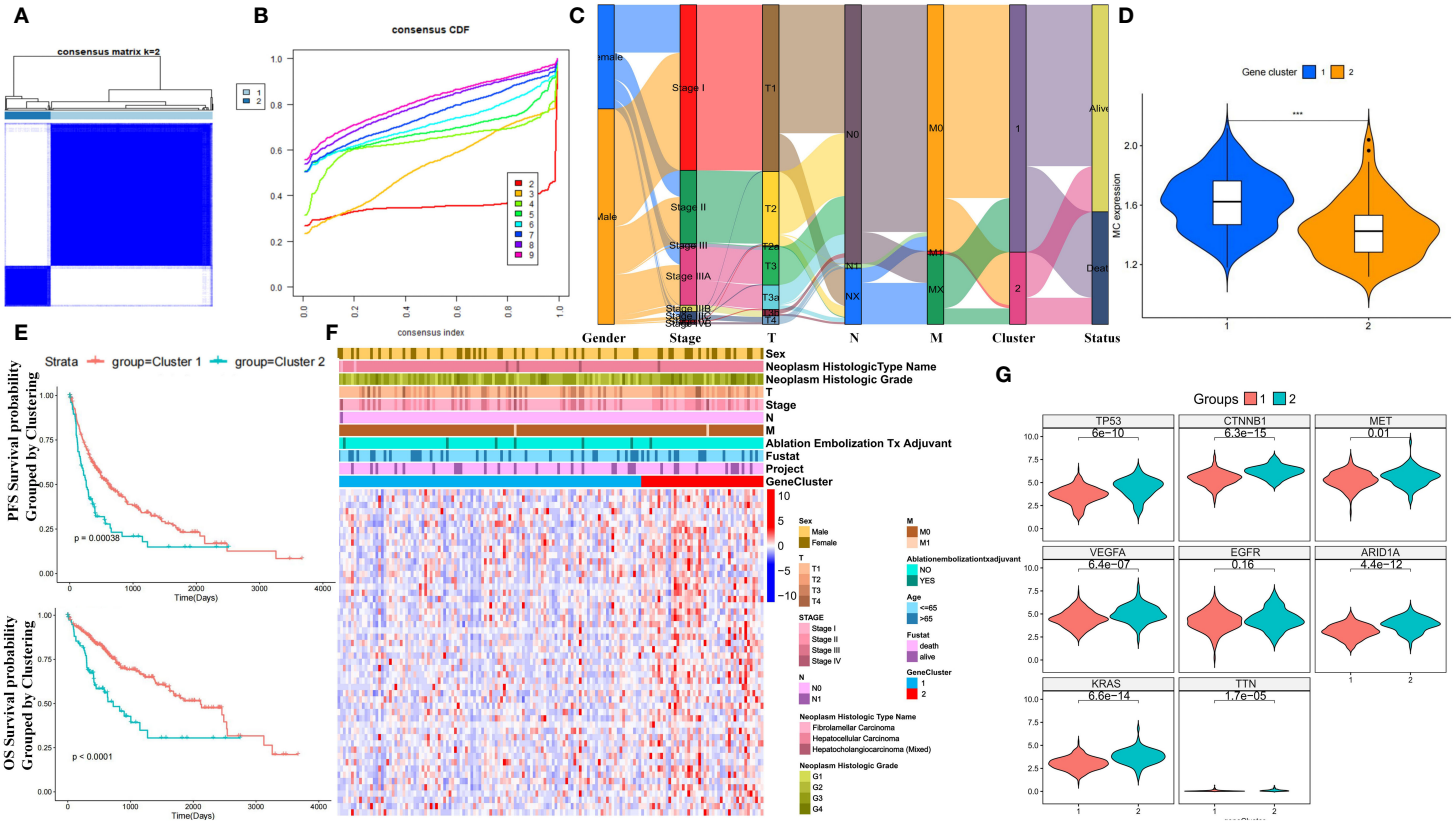
Prognostic value of unsupervised clusters based on MCGs in HCC. **(A)** Consensus matrices of HCC patients in the TCGA cohort for k = 2 using 1000 iterations of unsupervised consensus clustering method (K-means). **(B)** Consensus cumulative distribution function (CDF) plot showing clustering stability. **(C)** Alluvial diagram showing the changes of MC clusters, TNM stage and status. **(D)** Expression comparison of MC ssGSVA score between cluster 1 and 2 in HCC. **(E)** Kaplan-Meier curves for OS and PFS of TCGA cohort with the MC clusters in HCC. **(F)** Unsupervised clustering heatmap of all MCGs in TCGA cohorts with MC clusters, tumor stage, gender, age, stage, histologic grade, cancer type and status were used as patient annotations. **(G)** Expression comparison of Tumor-Related Genes between cluster 1 and 2 in HCC. (*** p < 0.001).

Unsupervised clustering of MCGs in HCC patients from TCGA identified two stable subtypes: cluster 1 (289 cases) and cluster 2 (80 cases) (**Figures 3A, B**). An Alluvial diagram showed that cluster 2 had progressed TNM stage and worse survival outcomes compared to cluster 1 (**Figure 3C**). Cluster 1 had higher MC ssGSVA scores and better survival prognosis in OS and PFS (log-rank test, P ≤ 0.05) (**Figures 3D, E**), although differences in DSS and DFI were not significant (**Supplementary Figure S5A**). Patients divided by MC ssGSVA score also showed significantly better prognosis in OS and PFS of high MC subgroup. Nevertheless, no significant differences were observed in terms of DSS and DFI survival prognosis (**Supplementary Figure S5B**). In the ICGC cohort, no statistically significant OS distinction was observed among patients stratified by MC ssGSVA score, though a trend towards varying survival outcomes was noted (log-rank test, P=0.08) (**Supplementary Figure S5C**). The heatmap of differentially expressed MCGs indicated that cluster 2 had higher expression of tumor-related genes and worse survival outcomes (**Figures 3F, G**). Cluster 1 was thus defined as the HCC subtype with higher MC levels and better prognosis, and cluster 2 with lower MC levels and worse prognosis. Next, we performed WGCNA on 94 MCGs and successfully divided them into 7 modules, as shown in Hierarchical clustering dendrogram and Modular-trait relationships (**Supplementary Figures S5D, E**). The blue module was highly correlated with low-MC (Cor = 0.48, P = 3e^−22^), in which a positive correlation between module membership and gene significance were observed (**Supplementary Figure S5F**). Genes in the blue module were significantly enriched in mitosis, cell cycle, mitotic nuclear division, spindle pole, and the p53 signaling pathway (**Supplementary Figures S5G, H**).

### 3.5 Machine learning for integrative construction of a consensus signature

**Figure 4:**
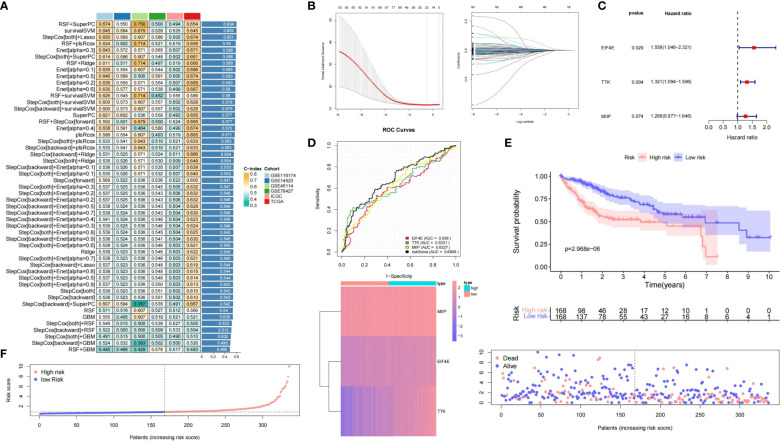
Construction and validation of prognostic MCGs-based signature for HCC. **(A)** A total of 50 kinds of prediction models via LOOCV framework and further calculated the C-index of each model across all validation datasets. **(B)** Selection of optimal λ in LASSO regression. **(C)** Univariate Cox regression of 3 core prognostic mRNAs. **(D)** Time-dependent ROC curves of the MCGs signature based on entire datasets. **(E)** Kaplan-Meier curves of high-risk and low-risk subgroups. **(F)** Risk score distribution, heatmap and survival status in entire HCC datasets.

Using MCGs mRNA expression profiles, we employed integrative machine learning to develop a consistent MC riskscore signature. In multiple datasets (TCGA-LIHC, ICGC-LIHC, GSE116174, GSE14520, GSE45114, and GSE76427), 50 prediction models were fitted using LOOCV, with RSF + SuperPC, survivalSVM, and stepCox + LASSO performing best, the latter being optimal in TCGA (**Figure 4A**). The optimal λ in LASSO regression was determined when the partial likelihood deviation was minimal (**Figure 4B**). Stepwise Cox regression on 24 candidate MCGs with non-zero LASSO coefficients identified three core prognostic mRNAs: MIIP, TTK, and EIF4E (**Figure 4C**). The riskscore formula was: riskscore = (0.2375 × MIIP expression) + (0.2785 × TTK expression) + (0.4439 × EIF4E expression) (**Supplementary Table S1**). ROC curves based on the entire datasets showed that the MCGs signature riskscore has relatively stable predictive efficacy for overall survival time (**Figure 4D**). A good performance was observed in prediction prognosis at 1 year (0.778), 3 years (0.760), and 5 years (0.690) (**Supplementary Figure S6A**). KM curves showed significantly worse prognosis for high-risk versus low-risk subgroups (P = 2.968e^-6^) (**Figure 4E**). **Figure 4F** displays the riskscore distribution, heatmap, and OS status of the MCGs signature in the TCGA-LIHC dataset.

### 3.6 The role of MC signature in predicting benefit from immunotherapy and other treatments

To compare our MCGs signature with existing HCC signatures, we analyzed 47 published liver cancer mRNA signatures (**Supplementary Table S2**). Our MCGs model ranked highly in predictive performance in the TCGA-LIHC dataset (**Supplementary Figure S6B**). Verification in multiple external datasets (ICGC, GSE116174, GSE76427) confirmed the MCGs signature’s predictive ability (**Supplementary Figure S6C**). While some models outperformed our signature, it was selected from 94 MC-related genes and reduced dimensionally by machine learning, enhancing its extrapolation potential. Analysis in the GSE104580 cohort showed lower MCGs riskscore in TACE-responsive HCC patients, with an ROC AUC of 0.748 for TACE responsiveness prediction (**Supplementary Figure S6D**). In the IMvigor210 cohort, patients with lower riskscores showed better responses to anti-PD-L1 treatment (**Supplementary Figure S7A**). In GSE202069, anti-PD-L1 treated HCC liver samples had significantly lower MCGs riskscores than untreated samples, with an AUC of 0.945 for predicting immunotherapy response (**Supplementary Figure S7B**). In the melanoma GSE91061 cohort (treated with anti-PD-L1 and anti-CTLA4 immunotherapy), patients with PR/CR had a lower risk score compared to those with SD/PD, although this difference was not statistically significant (**Supplementary Figure S7C**). In the GSE223201 cohort, the MCGs risk score showed a decreasing trend in lenvatinib-treated HCC liver cancer compared to untreated patients (**Supplementary Figure S7D**), which was also observed in the GSE153203 cohort (**Supplementary Figure S7E**). We confirmed that combined lenvatinib and anti-PD-L1 pembrolizumab treatment significantly reduced the MCGs risk score in HCC liver cancer (GSE148355) (**Supplementary Figure S7F**). Additionally, the MCGs risk score was significantly higher in liver tissue from patients who underwent partial liver resection or transplant compared to normal liver tissue, with a significant upward trend from normal to G1-G3 stage liver cancer (**Supplementary Figure S7F**). In the GSE116174 cohort, low-risk subtype patients had significantly higher survival times, although no significant differences were observed in the ICGC and GSE76427 cohorts (**Supplementary Figure S7G**).

### 3.7 Drug sensitivity responses in different MCGs subgroups

Differential expression analysis showed higher expression of MIIP, TTK, and EIF4A in tumors versus normal tissues across various cancers (**Supplementary Figure S8A**). In TCGA-LIHC, these genes increased with higher stage (**Supplementary Figure S8B**), consistent with our riskscore (except for Stage IV and M) (**Supplementary Figure S8C**). Immunohistochemistry confirmed higher protein expression in liver cancer versus normal liver tissue (**Supplementary Figure S8D**). We calculated IC50 values for common HCC chemotherapies and evaluated drug sensitivity differences between MC subtypes (cluster 1 and 2) and prognostic risk subgroups (high and low-risk). Cluster 2 (low-MC) had higher IC50 values for sorafenib, doxorubicin, and pentofluorouracil compared to cluster 1 (high-MC), with no difference for cisplatin, whereas the opposite was true for Cetuximab (**Supplementary Figure S8E**). Consistent results were observed between high-risk and low-risk groups (**Supplementary Figure S8F**).

### 3.8 Immune infiltration differences in MC clusters

The ssGSVA score analysis of 16 types of immune cells and 13 immune-related pathways between MC clusters revealed distinct immune infiltration patterns (**Supplementary Figure S9A**). Specifically, cluster 1 was enriched in immune/inflammatory pathways (such as human leukocyte antigen (HLA), cytotoxic, type I/II interferon response) and immune cells (DC, macrophages, NK, and Treg cells). Wilcoxon testing indicated higher expression levels of PD-1, PD-L1, CTLA4, HAVCR2, LAG3, and TIGIT in cluster 2 compared to cluster 1 (P<0.05) (**Supplementary Figure S9B**). Heatmaps showed significant enrichment in cell cycle pathways (spliceosome, cell cycle, homologous recombination) and liver cancer stem cell signaling pathways (Notch, Wnt) in cluster 1, while cluster 2 exhibited enrichment in metabolism and coagulation cascades (**Supplementary Figure S9C**). Using the ESTIMATE algorithm, cluster 2 demonstrated lower StromalScore, ImmuneScore, and ESTIMATEScore (**Supplementary Figure S9D**). TIDE analysis indicated higher infiltration of myeloid-derived suppressor cells (MDSCs) and cancer-associated fibroblasts (CAFs) in cluster 2. Additionally, cluster 2 had higher TIDE dysfunction scores compared to cluster 1, suggesting a greater likelihood of immune escape and poorer immunotherapy efficacy (**Supplementary Figure S9E**). Analysis of immune pathways favored by cluster 1 (HLA, cytotoxic, type I/II interferon response) showed higher cytokine expression levels in cluster 1 (**Supplementary Figure S10A**). GSEA enrichment analysis revealed significant enrichment for antigen processing/presentation, NK cytotoxicity, and T cell receptor signaling in cluster 1 (**Supplementary Figure S10B**).

### 3.9 Immune infiltration and tumor microenvironment analysis based on MCGs grouping

**Figure 5:**
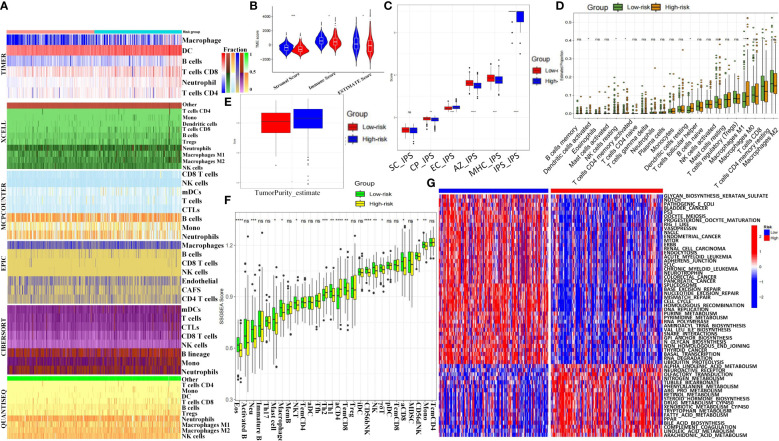
Immune landscape and therapeutic implications of MCGs signature in HCC. **(A)** Heatmap for immune infiltration based on TIMER, MCPCOUNTER, QUANTISEQ, XCELL, CIBERSORT, and EPIC algorithms among high and low risk subgroups. **(B)** Comparison of StromalScore, ImmuneScore and ESTIMATEScore between the risk subgroups. **(C)** Comparison of immunophenotypic scores between the risk subgroups. **(D)** Comparison of Immune cell proportions by CIBERSORT between the risk subgroups. **(E)** Comparison of tumor purity scores between the risk subgroups. **(F)** Comparison of ssGSVA scores of immune cells and pathways between the risk subgroups. **(G)** GSVA analyzed the key biological pathways of the two risk subgroups. (ns, not significant; p ≥ 0.05; * p < 0.05; ** p < 0.01; *** p < 0.001; **** p < 0.0001).

Heatmap analysis of immune infiltration percentages in high- and low-risk groups using six algorithms (TIMER, XCELL, MCPCOUNTER, EPIC, CIBERSORT, and QUANTISEQ) showed higher T cell and NK cell proportions in the low-risk group in most conditions (**Figure 5A**). Low-risk patients had higher StromalScore, ImmuneScore, and ESTIMATEScore (**Figure 5B**). Immunophenotypic score (IPS) analysis revealed significantly higher SC-IPS, CP-IPS, EC-IPS, AZ-IPS, MHC-IPS, and global IPS in the low-risk group (**Figure 5C**). CIBERSORT analysis showed more DC cells, T follicular helper cells, and M0 macrophages but fewer NK cells, CD8^+^ T cells, and M1 macrophages in the high-risk group (**Figure 5D**). Tumor purity score was also significantly higher in high-risk patients (**Figure 5E**). The ssGSVA scores of 28 immune cells and 13 pathways indicated lower immune infiltration in high-risk patients (**Figure 5F**, **Supplementary Figure S11A**). KEGG heatmaps revealed significant enrichment in tumor suppressor pathways (including p53, Mismatch Repair) and liver cancer stem cell signaling (Notch, Wnt) in the low-risk group, while the high-risk group was enriched in cancer-promoting pathways like tryptophan and phenylalanine metabolism (**Figure 5G**). Immune checkpoint expression analysis indicated significantly higher levels of LAG3, CD274, and HAVCR2 in the high-risk group (**Supplementary Figure S11B**). CTRP and GDSC drug sensitivity results identified compounds such as BHG712, AR-42, and BI-2536 as potential therapeutics (**Supplementary Figures S11C, D**). Drug target docking analysis for AR-42 and BI-2536 revealed stable binding with proteins except for BRCA1 (**Supplementary Figures S12A, B**). Lower IC50 values for AR-42 and BI-2536 were observed in high-risk patients (**Supplementary Figures S12C, D**).

### 3.10 Construction of nomogram for clinical diagnosis

**Figure 6:**
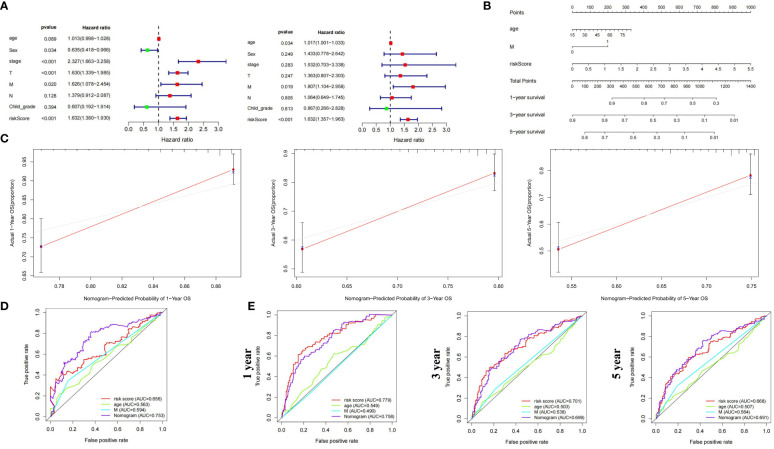
Construction and validation of prognostic nomogram based on MCGs signature for HCC. **(A)** Univariate and Multiple Cox regression of riskscore and traditional indicators in predicting HCC survival. **(B)** Nomogram based on MCGs signature in predicting prognosis of HCC patients. **(C)** Calibration chart valuating the survival probability of nomogram at 1, 3, and 5 years. **(D)** Time-dependent ROC curves of the nomogram based on entire dataset. **(E)** Time-dependent ROC curves of the nomogram at 1, 3, and 5 years.

We constructed a nomogram incorporating clinical indicators and riskscore to improve predictive performance. Candidate variables included MCGs riskscore, age, gender, Child-Pugh grade, Stage, and TNM stage. Univariate and multivariate Cox regression suggested age, M stage, and riskscore as independent prognostic factors for HCC (**Figure 6A**). These factors were combined to create a prognostic nomogram (**Figure 6B**). The calibration curve indicated the nomogram’s performance was comparable to the ideal model, with no significant deviation observed (**Figure 6C**). Survival ROC curves showed better overall and 5-year survival prediction with the nomogram compared to riskscore alone, while 1- and 3-year survival might be better predicted by MC riskscore alone (**Figures 6D, E**).

### 3.11 ScRNA-seq analysis reveals dynamic MCGs expression profiles in HCC

**Figure 7:**
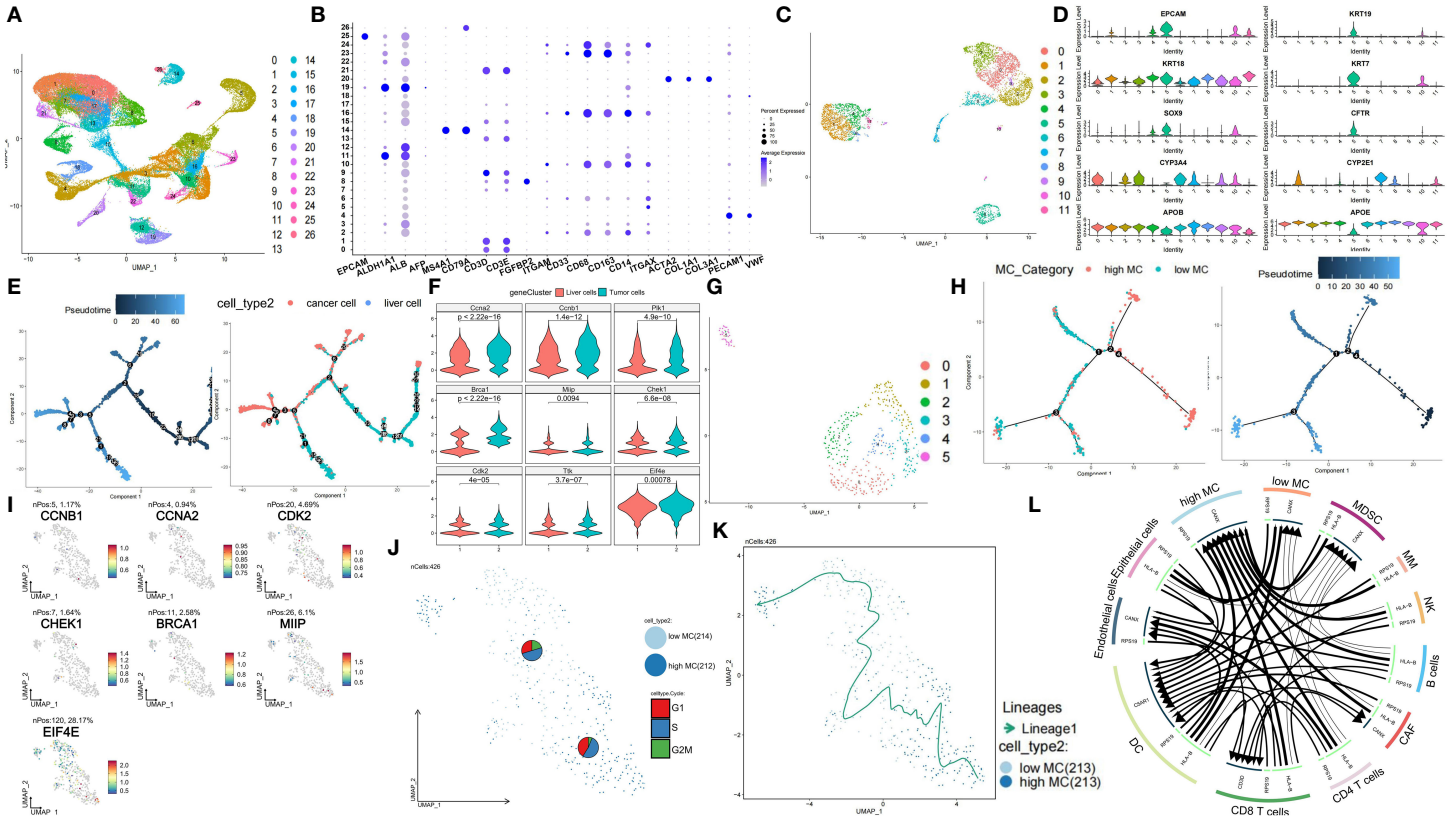
Single-cell characterization of cellular heterogeneity of MCGs in HCC. **(A)** UMAP clustering of diverse cell types and percentages in GSE202642. **(B)** Bubble plot of diverse cell types marker expression. **(C)** UMAP clustering of diverse tumor and liver cell types and percentages. **(D)** Violin plots of diverse tumor and liver cell types marker expression. **(E)** Pseudotime trajectory plot of single cells colored by pseudotime order and cell types. **(F)** Expression comparison of hub and prognostic MCGs between liver and tumor cells. **(G)** UMAP clustering of diverse tumor cell types and percentages. **(H)** Pseudotime trajectory plot of single cells colored by pseudotime order and high/low MC cell types. **(I)** PAGA feature plots of MCGs expression. **(J)** PAGA plot of single cells with cycle phase proportion, stratified by cell types. **(K)** Trajectory inference of single cells grouped by high/low MC type. **(L)** Inter-cell communication network between high/low tumor cells and immune cells.

We obtained scRNA-seq data comprising 74,957 cells from 7 HBV-related HCC tissues from the GSE202642 dataset. The quality control results are displayed in **Supplementary Figures S13A** and **B**. We then normalized the data and performed SCTransform analysis to identify highly variable genes (**Supplementary Figure S13C**). Next, we conducted PCA dimension reduction. The PCA components, eigengenes, and corresponding heatmaps are shown in **Supplementary Figures S13D** and **E**. The Elbow Plot revealed a clear inflection point at PC=13, so we included the first 13 principal components for subsequent analysis (**Supplementary Figure S13F**). UMAP clustering analysis clustered the total single cell data (**Figure 7A**) and EPCAM, MS4A1, CD79A, FGFBP2, CD68, ACTA2, PECAM1 marker gene plots identified cell types (**Figure 7B**, **Supplementary Figures S14A, B**). Heatmap of key genes and KEGG pathway in differential clusters of solid cells and immune cells were shown in **Supplementary Figure S14C**. Normal liver cells and tumor cells clusters were extracted for separate UMAP clustering (**Figure 7C**) and further classified by AFP, PTPRC, ALB, GPC3 (**Figure 7D**). Pseudotime analysis showed significant temporal sequence from hepatocytes to tumor cells (**Figure 7E**) with significant MCGs differences (**Figure 7F**). Then, tumor cells were extracted and divided into 6 clusters (**Figure 7G**). Marker genes of each cluster were shown in **Supplementary Figure S14D**. Tumor cells were divided into high and low MC subgroups by ssGSVA score. Pseudotime analysis revealed cell differentiation trend from high to low MC tumor cells (**Figure 7H**). Among 6 functional and 3 prognostic MCGs, CCNA2, CCNB1, CHEK1, BRCA1, MIIP and EIF4E significantly increased from hepatocytes to HCC (**Supplementary Figure S14E**). From high/low MC bifurcation point 2, CCNA2, CCNB1, MIIP and EIF4E significantly increased (**Supplementary Figure S14E**). PAGA analysis visualized data of liver cancer single cells and marker genes (**Supplementary Figures S14F, G**). Since PLK1 and TTK had almost no expression, the remaining 5 functional and 2 prognostic MCGs were compared between high/low MC tumor cells (**Figure 7I**). Heatmaps of phase percentages, cycle scores, and MCGs expression suggested both MC-subtype tumor cells were mainly in S phase, while low-MC tumor cells had higher G2M versus G1 percentage, suggesting greater proliferation (**Figure 7J**, **Supplementary Figure S14H**). The PAGA trajectory analysis results were consistent with the pseudotime analysis (**Figure 7K**), indicating that high MC tumor cells differentiate into low MC tumor cells. Inter-cellular communication analysis revealed significantly less communication between low-MC tumor cells and immune cells versus high-MC tumor cells (**Figure 7L**).

### 3.12 ST analysis reveals spatial differences of high and low MC tumor tissue in HCC

**Figure 8:**
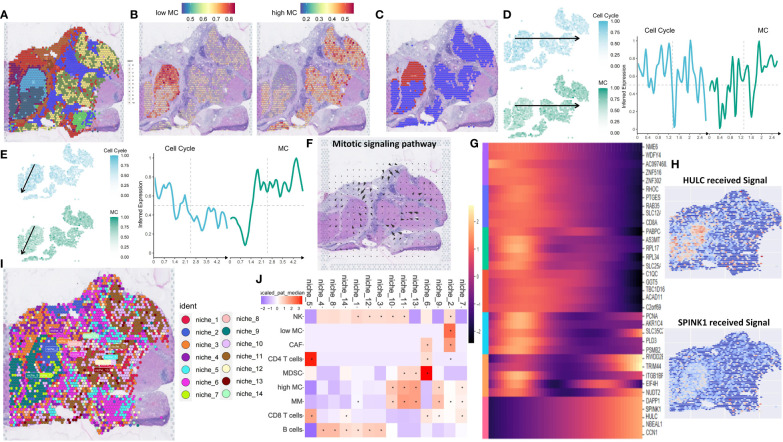
Spatial transcriptomic analysis reveals intratumor heterogeneity of MCGs in HCC. **(A)** Spatial UMAP clustering of liver parenchyma region and localization of cell clustering patterns. **(B)** Integration with low and high MC scRNA-Seq cell annotations. **(C)** Spatial localization of high MC and low MC tumor spots. **(D)** Change of z-scored cell cycle and MC score along the Horizontal spatial trajectory. **(E)** Change of z-scored cell cycle and MC score along the vertical spatial trajectory. **(F)** Mitotic signaling direction in human liver cancer tissue. **(G)** Identification of the differentially expressed genes due to the total amount of received signal in the Mitotic signaling pathway. **(H)** Identification of the differentially expressed genes due to the total amount of received signal in the Mitotic signaling pathway. **(I)** Schematic of cell-type niche definition and UMAP of spatial transcriptomics spots based on cell-type compositions. **(J)** Scaled median cell-type compositions within each niche.

ST analysis of this HCC sample showed higher total RNA expression in the left tumor region (**Supplementary Figure S15A**). Data processing included SCTransform normalization and PCA dimension reduction (**Supplementary Figures S15B, C**). UMAP clustering identified 11 clusters (**Figure 8A**). Gene-set enrichment analysis based on the XCELL method showed lower immune pathway scores (HLA, cytotoxic, type I/II interferon response) in the left tumor region compared to the right region (**Supplementary Figure S15D**). The left region was defined as an immune-poor region (yellow dashed outline) and the right region as an immune-rich region (black dashed outline) (**Supplementary Figure S15E**). Immune-poor regions were represented by ST clusters 4 and 6, while immune-rich regions were represented by ST clusters 2 and 3 (**Figure 8A**). MC scores were enriched in the immune-rich region, and cell cycle scores were enriched in the immune-poor region (**Supplementary Figure S15F**).

Using the scRNA-seq dataset (GSE202642) as a reference, we mapped the distribution patterns of low and high MC tumor cells in each region. Low MC tumor cells were primarily located in the upper portion of the immune-poor area, corresponding to ST cluster 6, while high MC tumor cells were found in ST clusters 2, 3, and 4 (**Figures 8B, C**). Using the SPATA package, we examined tumor heterogeneity’s spatial distribution. MC scores decreased, and cell cycle scores increased from low to high MC regions (**Figures 8D, E**). Similar trends were observed for hub and prognostic MCGs (**Supplementary Figures S15G, H**).

Spatial signaling flow analysis using the COMMOT package revealed mitotic signals converging towards the low MC region (**Figure 8F**). Tumor proliferation pathways like TGF and TNF were directed to the low MC region, while the tumor suppressor stimulator of interferon genes (STING) pathway flowed to the high MC region, implicating potential tumor recurrence (**Supplementary Figure S15I**). Heatmap analysis of differential downstream genes of mitotic signals showed increased SPINK1 and HULC expression in the low MC region (**Figures 8G, H**).

**Figure 9:**
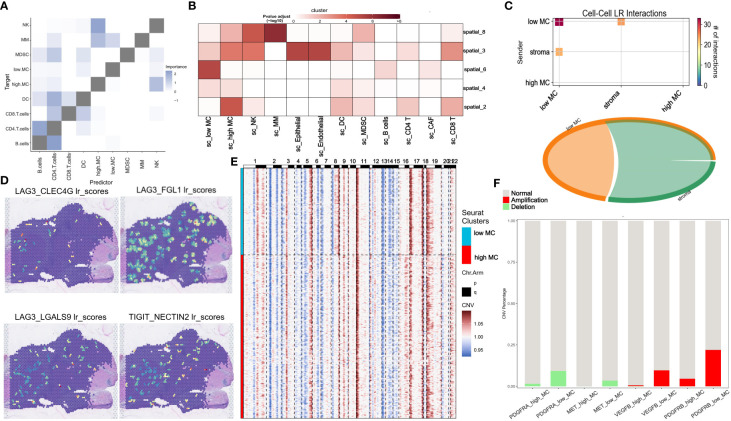
Spatial transcriptomic analysis reveals immune escape-related signaling and intra-cellular LP interactions in HCC. **(A)** Median importance of cell-type abundance in the prediction of abundances of other cell types within a spot. **(B)** The MIA map of all scRNA-seq-identified cell types and ST-defined regions. **(C)** Inter-cell communication network of immune escape signaling between high/low MC tumor spots and stromal spots. **(D)** ligand-receptor scores of LAG3_FGL1, LAG3_CLEC4G1, LAG3_LGLS92 and TIGHT_NECTIN2 in human liver cancer tissue. **(E)** CNV analysis for low and high MC spots. **(F)** Proportion of CNV amplification and deletion of specific genes.

To further understand spatial interactions within liver cancer tissues, unsupervised clustering of regions by cell type composition identified nine representative spot microenvironments, named NICHES (**Figure 8I**). Contrasted with NICHES 1, 7, 9, 10 and 11, which contained high MC tumor cells and macrophages. NK cells were abundant in NICHES 10 and 11, while CD8^+^ T cells were more prevalent in NICHES 7 and 9 (**Figure 8J**). The low MC region had minimal adjacent immune cells, whereas the high MC region had significant NK cell abundance (**Figure 9A**, **Supplementary Figure S16A**). The MIA method indicated significant overlap between specific genes of low MC tumor cells and CAFs with the cancer region-specific genome of ST cluster 6. In contrast, low MC tumor cells and immune cells (NK cells, CD4^+^ T, CD8^+^ T) overlapped significantly with ST clusters 2 and 3 (**Figure 9B**).

Using the stLearn SCTP algorithm, unsupervised analysis uncovered significant immune escape-related ligand-receptor (LR) interactions within the low MC region and between the low MC region and the stromal region, with CD96-PVR showing the most significant difference (**Supplementary Figure S16B**). The cell-cell interaction chord map revealed minimal immune escape-related LR interactions in the high MC region (**Figure 9C**). LAG3-CLEC4G, LAG3-FGL1, LAG3-LGALS9, and TIGHT-NECTIN2 were enriched in the low MC region (**Figure 9D**). Mitotic LRs were also enriched in the low MC region (**Supplementary Figure S16C**).

**Figure 10:**
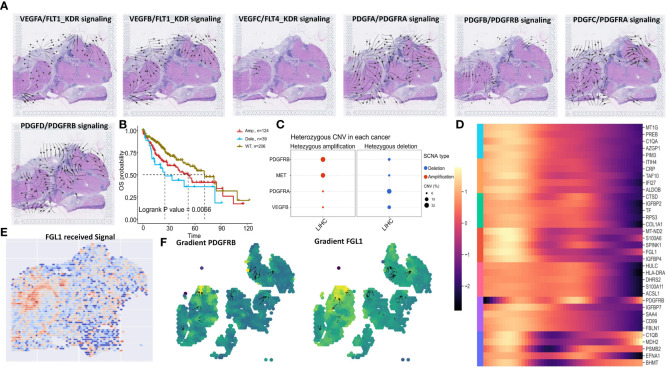
Spatial transcriptomic analysis reveals PDGFRB signaling and Genomic Alterations in HCC. **(A)** VEGFA/FLT1_KDR, VEGFB/FLT1_KDR, VEGFC/FLT4_KDR, PDGFA/PDGFRA_PDGFRB, PDGFB/PDGFRA_PDGFRB, PDGFC/PDGFRA, PDGFD/PDGFRB signaling direction in human liver cancer tissue. **(B)** OS of PDGFRB CNV in TCGA LIHC. **(C)** Heterozygous amplification and deletion of specific genes CNV in TCGA LIHC. **(D)** Identification of the differentially expressed genes due to the total amount of received signal in the Mitotic signaling pathway. **(E)** The level of FGL1 received signal. **(F)** Vector fields consisting of the aligned gradients of spatial shifts in gene-expression levels.

Considering tumor recurrence after CABO/NIVO treatment may relate to genetic mutations, subclonal CNV mapping revealed differences between low and high MC regions (**Figure 9E**, **Supplementary Figure S16D**). Among major CABO/NIVO immune pathway genes (PD1/PD-L1, VEGF, PDGF, MET), PDGFRA, PDGFRB, VEGFB, and MET exhibited significant deletions/amplifications between low and high MC regions (**Supplementary Figure S16E**, **Figure 9F**). Spatial signaling analysis indicated VEGF and PDGF signaling towards low MC areas (**Figure 10A**), while HGF/MET and PD1/PDL1 pathways did not (**Supplementary Figure S16F**). PDGFRB deletion/amplification was associated with reduced survival in liver hepatocellular carcinoma patients (**Figure 10B**). Heterozygous CNV mapping showed frequent PDGFRB amplification (**Figure 10C**). Analysis of PDGFB/PDGFRB, and PDGFD/PDGFRB pathway differential downstream genes revealed increased FGL1 expression but decreased CPR, TF, and HLA-DRA expression (**Figure 10D**), consistent with spatial region differential expression (**Figure 10E**, **Supplementary Figure S16G**). PDGFRB and FGL1 exhibited similar spatial vector field trajectories (**Figure 10F**).

## 4 Discussion

In this study, we verified that the MC pathway plays a pivotal role in HCC progression and has potential in regulating the TME (6). Six MC hub genes and three MC prognostic genes exhibited significant temporal and spatial differences during HCC development. Single-cell and ST analysis revealed the distribution of tumor cells/regions with varying MC levels in specific clusters or tissue regions, with immune deserts in the low MC region potentially triggering immune escape. Targeting the MC pathway may be a promising HCC treatment approach. The constructed nomogram combining MC riskscore and clinical indicators enables personalized clinical decision-making. Top-scoring MC drug-sensitive small molecules like BI-2536 and AR-42 were identified to inhibit HCC progression by targeting MCGs via protein molecular docking.

MC represents a precursor to apoptosis or necrosis, where improper cell division causes DNA damage and abnormal chromosome segregation, ultimately inducing MC as a tumor suppression mechanism (6, 8). While some chemotherapy drugs effectively prompt MC at low doses (e.g., fluorouracil, doxorubicin) (11, 12, 55), few studies have bioinformatically evaluated the immune effect of MC due to the high HCC heterogeneity and complex escape mechanisms. We aimed to explore specific mechanisms of MC in HCC via high-throughput sequencing and identify MC-related protein targets for drug development.

The American Joint Committee on Cancer (AJCC) staging system has long been used to evaluate tumor progression and guide treatment but is limited by tumor heterogeneity and individual differences. HCC treatments have diversified (TACE, anti-angiogenics, PD-L1 inhibitors, multi-kinases), yet reliable predictive biomarkers are still lacking (56–59). This study integrated 10 single and 50 combination machine learning models to test six HCC bulk RNA-sequencing datasets (40). The LASSO+MultiCOX model showed great predictive value, especially for TCGA-LIHC data. ROC analysis revealed that the MC prognostic model has high accuracy and stability. Although past clinical practice utilized TNM staging and AJCC staging reliability (60), our MC features are independent with better performance, ranking well cross-datasets compared to 47 other cancer features. MCGs protein overexpression, as per the Human Protein Atlas (HPA) and GSCA databases, associates with poorer prognosis. A model combining age, M stage, and MC riskscore better predicted 5-year survival than individual signatures, potentially serving as a new clinical evaluation indicator.

This study confirmed that high-risk HCC patients with low MC demonstrate higher sensitivity to three chemotherapy drugs (sorafenib, doxorubicin, fluorouracil). Sorafenib is the classic first-line unresectable HCC drug (13, 59), selectively inducing apoptosis via Bcl-2/caspase-3 while inhibiting normal liver cell cycle (13). Drugs inactivating p53, like doxorubicin, also rapidly prompt MC (61, 62). P53 exhibited the highest mutation level among all our MCGs here. In summary, integrating MC status with existing clinical indicators may better guide precision treatment. Further exploration of spatial MC heterogeneity mechanisms could yield immunotherapeutic targets against HCC immune escape.

Cancer immunotherapy, represented by immune checkpoint inhibitors (ICIs) like PD-L1 inhibitors (nivolumab) and anti-angiogenics (bevacizumab), has transformed solid tumor treatment as a new strategy (63, 64). With proven efficacy, the former received FDA approval as a second-line advanced liver cancer therapy. The CheckMate 040 trial showed that the PD-L1 inhibitor nivolumab achieved promising first-line advanced liver cancer results, although the phase III trial did not significantly improve overall survival (65). We found that anti-PD-L1 monoclonal antibodies alone or combined with lenvatinib can significantly reduce HCC patient MC riskscore. Studies suggest that ICI response in mismatch repair deficient/microsatellite unstable colorectal cancer may result from decreased CD8^+^ resident memory T cell (Trm) mitosis and increased proportions of immune-activated cells (CD8^+^ effector memory T cells (Tem), CD4^+^ helper T cells (Th), CD20^+^ B cells) (66). However, one study showed heightened high mitosis level and metastasis/spread risk after TACE (67), suggesting a complex underlying mechanism given our model’s accurate TACE response prediction.

Interestingly, MC also mediates acquired drug resistance. Chemotherapy activates pro-survival pathways causing resistance. Continued DNA damage repair inhibition combined with therapy can counter this. While several chemotherapeutic drugs (inhibitors of Aurora kinase, CHK1, Polo-like kinases (PLKs), survivin, and kinesin-related proteins) induce MC at lower doses, the resulting tetraploid or aneuploid tumor cells are resistant to mitotic abnormalities. Mutational sensitivity also contributes to increased drug sensitivity (6, 68). Small molecules have attracted attention due to diverse chemical properties and biological activities. Genomics of Drug Sensitivity in Cancer (GDSC) and Cancer Therapeutics Response Portal (CTRP) analysis revealed MC hub/prognostic genes show highest sensitivity towards two compounds - BI-2536 (PLK1 inhibitor) and AR-42 (histone deacetylase (HDAC) inhibitor). Clinical trials found most elderly relapsed/refractory acute myeloid leukemia (AML) patients treated with BI-2536 displayed characteristic mitotic arrest with increased G2/M bone marrow cells. We evaluated MCG binding to BI-2536/AR-42, finding hydrogen bonds and electrostatic interactions enable stable binding. The combination of low binding energy and stability explains the prediction of high sensitivity among high MC risk patients to these drugs.

Increasing evidence shows that mitotic differences in tumor cells are regulated by immune infiltration, in turn affecting the TME immune status (55, 69). Studies have shown high-proliferative triple-negative breast cancer can prompt homologous recombination defects and genomic instability, while low-proliferative tumors enrich for mitosis-related genes (70). Tetraploidy from MC imbalance can induce DNA damage responses, destabilize genomes, and lead to aggressive tumors with immune evasion and drug resistance (55). Our results demonstrate that HCC patients with low MC show reduced immune infiltration and poorer prognosis. Further analysis revealed lower MC expression significantly associates with lower NK and T cell levels.

MC is a complex cellular event regulated by multiple factors and may be affected by heterogeneity and batch effects in batch sequencing. Single-cell and ST analysis can effectively analyze TME mutual regulation. We utilized these technologies to study MC-related gene expression in liver cancer and connections with tumor cells and TME. MCGs demonstrate temporal and spatial distribution heterogeneity. Meanwhile, low MC tumor cells appear to malignantly proliferate from high MC tumor cells. Consequently, communication with immune cells was significantly reduced, aligning with previously reported MC roles in driving proliferation and impacting the TME (16, 45, 69). We then deeply analyzed the complex TME-MC relationship. The MC distribution in liver cancer tissues highly coincides with previously reported immune-rich/immune-depleted regions (48), highlighting an important MC-immune connection. Meanwhile, mitotic signal enrichment in low-MC regions suggests a potential regulatory role for MC in cell cycle progression. Each signaling pathway also exhibited specific distribution patterns in the spatial microenvironment, with mitotic signals, tumor necrosis factor-gamma (TNG-γ), and transforming growth factor-beta (TGF-β) pathways showing consistent spatial flow directionality towards low MC areas. In contrast, the STING pathway preferentially localized to high MC regions. Activating cyclic GMP-AMP synthase (cGAS)-STING signaling may alleviate DNA damage and induce MC and innate immune activation, improving responses to immune checkpoint blockade (ICB) in solid tumors (71).

We found immune cells and pathways (HLA, cytotoxic, type I/II interferon response) were significantly reduced in low MC regions, with significantly different immune cell compositions between high and low MC regions. Niche clustering and proximity analysis revealed CAFs enrichment with low MC tumor cells, while almost no CD4^+^ T cell distribution. CAFs can promote tumor cell mitotic proliferation via PDGFC/PDGFRA/SLUG, achieving metastasis and immune escape by regulating E2F, signal transducer and activator of transcription 5 (STAT5), etc. (72, 73). The niche with high MC tumor cells exhibited diverse immune infiltrates. MistyR analysis further supports these conclusions, with almost no adjacent immune cells in low MC regions yet abundant proximate NK cells adjacent to cancer cells in high MC regions, which reveals potential MC signaling, immune microenvironment, and HCC recurrence interactions. The spatial distribution of immune escape-related genes in liver cancer reveals low MC tumor areas may exhibit immune escape by activating such pathways. Combined with CNV hypervariable gene and COMMOT signal flow analysis of key CABO/NIVO targets, we found PDGFRB amplification mutations may be a key factor triggering downstream signaling cascades. PDGFRB mutations associate with tumor immune evasion (74). Dermawan et al. also reported PDGFRB mutations and diffuse overexpression in undifferentiated malignant epithelioid tumor clinical samples (75). The enrichment of PDGFRB mutations in low MC density regions suggests a potential mechanistic link, revealing MC-induced regulation of PDGFRB alterations. The PDGF/PDGFRB signaling we observed in the low MC region activates the downstream lymphocyte-activation gene 3 (LAG3)/fibrinogen-like protein 1 (FGL1) immunosuppressive axis, adding complexity to liver cancer immune evasion analysis. LAG3 combined with liver-derived FGL1 can inhibit T cell activity, with FGL1 knockout enhancing T cell responses (76). LAG3/FGL1 signaling also associates with reduced immunotherapy sensitivity and acquired resistance in tumors (76, 77). Therefore, activation of this axis in low MC areas indicates region-specific immune regulation and may partially explain the role of spatial MC heterogeneity in driving tumor recurrence.

Our study has some limitations. First, our cohort was derived from different sequencing platforms and databases with inevitable differences in gene annotation and tumor heterogeneity. Second, the complex TME-tumor cell interaction mechanisms in high/low MC region contexts need further elucidation through extensive fundamental research. Finally, all our samples were retrospective and therefore the prognostic efficacy and immunotherapy response of MC in HCC should be prospectively evaluated in a multicenter cohort.

## 5 Conclusions

In summary, our integrated transcriptomics analysis reveals that MC heterogeneity significantly impacts HCC progression and therapeutic response. We identify an MCGs prognostic signature in HCC. Spatial mapping further associates low MC tumor regions with immune escape, mediated by PDGFRB signaling activating the downstream immunosuppressive LAG3/FGL1 axis. Elucidating the interplay between aberrant MC and immunity provides context to bulk sequencing interpretation and resistance mechanisms.

## Data availability statement

The original contributions presented in the study are included in the article/**Supplementary Material**. Further inquiries can be directed to the corresponding authors.

## Author contributions

ZM: Writing – review & editing, Writing – original draft, Visualization, Validation, Resources, Methodology, Investigation, Funding acquisition, Formal analysis, Data curation. ZG: Writing – original draft, Conceptualization, Software, Project administration, Investigation, Formal analysis, Data curation. RL: Writing – original draft, Validation, Software, Project administration, Investigation, Conceptualization. HG: Writing – review & editing, Project administration, Methodology, Investigation, Data curation. LC: Data curation, Validation, Visualization, Writing – review & editing. SH: Data curation, Methodology, Formal analysis, Validation, Visualization, Software, Writing – review & editing. GY: Data curation, Methodology, Supervision, Conceptualization, Formal analysis, Project administration, Validation, Investigation, Resources, Visualization, Software, Writing – original draft, Writing – review & editing.

## Supplementary material

Supplementary Figure 1 Study design and analytical workflow.

Supplementary Figure 2 Examination of genomic variants of MCGs. **(A)**, TMB distribution across cancer types. **(B)**, Mutual exclusivity and co-occurrence analysis of the top 20 mutation MRGs. **(C)**, Visualization of MCGs variant allele frequencies in HCC. **(D)**, Signaling pathways enrichment analysis of the mutation MCGs in HCC. **(E)**, Summary of the survival difference between WT and mutation MCGs in the Pan-cancer.

Supplementary Figure 3 Impact of MCGs genomic status on prognosis and tumor microenvironment. **(A)**, Summary of the difference of immune infiltration between MCGs SCNA groups. **(B)**, Summary of the difference of immune infiltration between MCGs mutation groups. **(C)**, OS, DSS, DFI, PFS survival curve between MCGs WT and mutation in HCC. **(D)**, OS, DSS, DFI, PFS survival curve between MCGs WT and SCNA in HCC. **(E)**, Summary of the survival difference between WT and mutation MCGs in the Pan-cancer.

Supplementary Figure 4 Transcriptomic profiles of MCGs in cancer pathways and prognosis. **(A)** Summary of effect from MCGs mRNA on cancer pathway activity. **(B)** Summary of the survival difference between low and high MC groups in the Pan-cancer. **(C)** Box plot comparing the MCGs ssGSVA score between tumor and normal samples in the Pan-cancer. **(D)** Summarized association between MCGs ssGSVA score and activity of cancer related pathways in HCC. **(E)** Association between MCGs ssGSVA score and immune cells infiltrates in HCC. **(F)** GSEA enrichment analysis of MCGs in HCC.

Supplementary Figure 5 Validation of prognostic value of MCGs clusters. (A), Kaplan-Meier curves for DSS and DFI of TCGA cohort with the MC clusters in HCC. **(B)**, Kaplan-Meier curves for OS, DSS, DFI, PFS of TCGA cohort with the low/high MCGs ssGSVA score in HCC. **(C)**, Kaplan-Meier curves for OS of ICGC cohort with the low/high MCGs ssGSVA score in HCC. **(D)**, Hierarchical clustering dendrogram of genes based on gene co-expression analysis. (E), Correlation of gene significance for MC and membership in blue module. **(F)**, Correlation of blue module membership with gene significance for MC. **(G)**, GO enrichment of mRNAs in blue module. **(H)**, KEGG enrichment of mRNAs in blue module.

Supplementary Figure 6 Validation of predictive performance of MCGs signature in multiple datasets. **(A)**, Time-dependent ROC curves of the MCGs signature at 1, 3, 5 years. **(B)**, Comparison of prediction performance of MCGs signature with published signatures in TCGA. **(C)**, Comparison of prediction performance of MCGs signature with published signatures in multiple validation datasets. **(D)**, Comparison of prediction of MCGs signature in the GSE104580 cohort.

Supplementary Figure 7 Relevance of MCGs signature to tumor drug response and progression. **(A)**, Comparison of prediction of MCGs signature in anti-PD-L1 response, and Kaplan-Meier curves of high-risk and low-risk subgroups in the IMvigor210 cohort. **(B)**, Comparison of MCGs ssGSVA score in anti-PD-L1 response in the GSE202069 cohort. **(C)**, Comparison of prediction of MCGs signature in anti-PD-L1/CTLA4 response in melanoma in the GSE91061 cohort. **(D)**, Comparison of prediction of MCGs signature in Lenvatinib response in the GSE223201 cohort. **(E)**, Comparison of prediction of MCGs signature in anti-PD-L1 and Lenvatinib response in the GSE153203 cohort. **(F)**, Comparison of MCGs ssGSVA score in differential stage of HCC tissue in the GSE5148355 cohort. **(G)**, Kaplan-Meier curves of high-risk and low-risk subgroups in the GSE116174, ICGC, GSE76427 cohort.

Supplementary Figure 8 Expression of prognostic MCGs in tumor progression and drug sensitivity. **(A)**, Comparison of mRNA expression of prognostic MGCs between normal and tumor in the Pan-cancer. **(B)**, Comparison of mRNA expression of prognostic MGCs in differential HCC stage. **(C)**, Comparison of risk score of MCGs signature of differential TNM staging, T staging, N staging, M staging and Child_Pugh staging. **(D)**, Comparison of protein expression of prognostic MGCs between normal and tumor tissue. **(E)**, The estimated half-maximal inhibitory concentration (IC50) of Sorafenib, Doxorubicin, Cetuximab, 5-Fluorouracil, and Cisplatin for response between the two clusters. **(F)**, The estimated half-maximal IC50 of Sorafenib, Doxorubicin, Cetuximab, 5-Fluorouracil, and Cisplatin for response between the two risk subgroups.

Supplementary Figure 9 Association of MCGs clusters with tumor microenvironment and immune profiles. **(A)** enrichment scores of 16 types of immune cells and 13 immune-related pathways between the two clusters. **(B)** The expression of immune checkpoint molecules in the two clusters. **(C)** GSVA analyzed the key biological pathways of two clusters. **(D)** Comparison of StromalScore, ImmuneScore and ESTIMATEScore between the two clusters. **(E)** Comparison of TIDE Score between the two clusters.

Supplementary Figure 10 Examination of immune characteristics of MCGs clusters. **(A)**, Gene expression in cytotoxic, HLA and type I/II interferon response pathway gene sets between two clusters. **(B)**, GSEA of immune-associated biological processes in the TCGA-LIHC cohort.

Supplementary Figure 11 Examination of immune and drug sensitivity association of MCGs signature. **(A)**, enrichment scores of 13 immune-related pathways between the two risk subgroups. **(B)**, The expression of immune checkpoint molecules in the risk subgroups. **(C, D)** Drug sensitivity analysis of compound targeted prognostic MGCs in the GDSC and CTRP.

Supplementary Figure 12 Prediction of MCGs-targeted therapy response by molecular docking. **(A)**, Binding mode of BI-2536 to TTK, EIF4E, PLK1, CDK2, BRCA1 and CHEK1 by molecular docking. **(B)**, Binding mode of AR-42 to TTK, EIF4E, PLK1, CDK2, BRCA1 and CHEK1 by molecular docking. **(C, D)** The estimated half-maximal IC50 of BI-2536 and AR-42 for response between the two risk subgroups.

Supplementary Figure 13 Quality control and preprocessing of single-sell data. **(A)**, Violin plots of quality control metrics in GSE202642. **(B)**, Scatter plot between RNA counts and percentage of mitochondrial genes. **(C)**, Variable feature ranking plot after selection based on variance. **(D)**, PCA dimension loading plots showing top contributing genes for the first 4 principal components. **(E)**, Heatmap of PCA dimensions showing contributions of top genes to the first 4 principal components. **(F)**, Elbow plot showing percent variance explained by principal components.

Supplementary Figure 14 Analysis of single-Cell MCGs expression patterns. **(A)**, Bubble plot of diverse solid cells types marker expression. **(B)**, Bubble plot of diverse immune cells types marker expression. **(C)**, Heatmap of key genes and KEGG pathway in differential clusters of solid cells and immune cells. **(D)**, Heatmap of key genes in differential clusters of tumor cells. **(E)**, MCGs expression dynamics along branched pseudotime trajectory colored by high/low MC cells and pseudotime trajectory heatmap. **(F)**, PAGA feature plots showing high/low MC cell types. **(G)**, PAGA feature plots showing tumor-related gene expression. **(H)**, Combined heatmap of MCGs expression, phase proportions, and cycle score between high/low cell types.

Supplementary Figure 15 Quality control and tumor spatial heterogeneity analysis. **(A)**, Violin and spatial plots of number of expressed genes per spot reveal broad range of expression profiles across tissue. **(B)**, Group correlation plot of SCTransform and log normalization. **(C)**, PCA elbow plot and dimensionality reduction plot indicate optimal PCA dimensions for downstream analysis. **(D)**, X-cell scores of specific immune pathway in Spatial tumor spots. **(E)**, Definition of immune-rich region (black dashed) and immune-poor region (yellow dashed). **(F)**, X-cell scores of MC and Cell cycle pathway in Spatial tumor spots. **(G)**, Change of z-scored MCGs expression along the Horizontal spatial trajectory. **(H)**, Change of z-scored MCGs expression along the vertical spatial trajectory. **(I)**, TNF, TGF, STING signaling direction in human liver cancer tissue.

Supplementary Figure 16 Immune escape-related ligand and receptor signaling analysis in HCC spatial transcriptome. **(A)**, Visualization of cell-type abundance correlation. **(B)**, Scatter plot highlighting the top predicted LR pair by stLearn SCTP. **(C)**, ligand-receptor scores of CCNA2_CDK1, CCNA2_CDK2, CCNB1_CDK1 and CCNB2_CDK1 in human liver cancer tissue. **(D)**, Line Plot Visualization of CNV Analysis. **(E)**, The volcano map showing differences in gene CNV amplification and deletion. **(F)**, HGF/MET signaling and PD1/PDL1 signaling direction in human liver cancer tissue. **(G)**, The level of received signal are shown for CRP, TF and HLA-DRA signaling.
