# Supplementary figures and images for: Mitotic catastrophe heterogeneity: implications for prognosis and immunotherapy in hepatocellular carcinoma
Source: Front Immunol. 2024 Jul 1;15:1409448. doi: 10.3389/fimmu.2024.1409448 (PMC11250588; doi:10.3389/fimmu.2024.1409448)

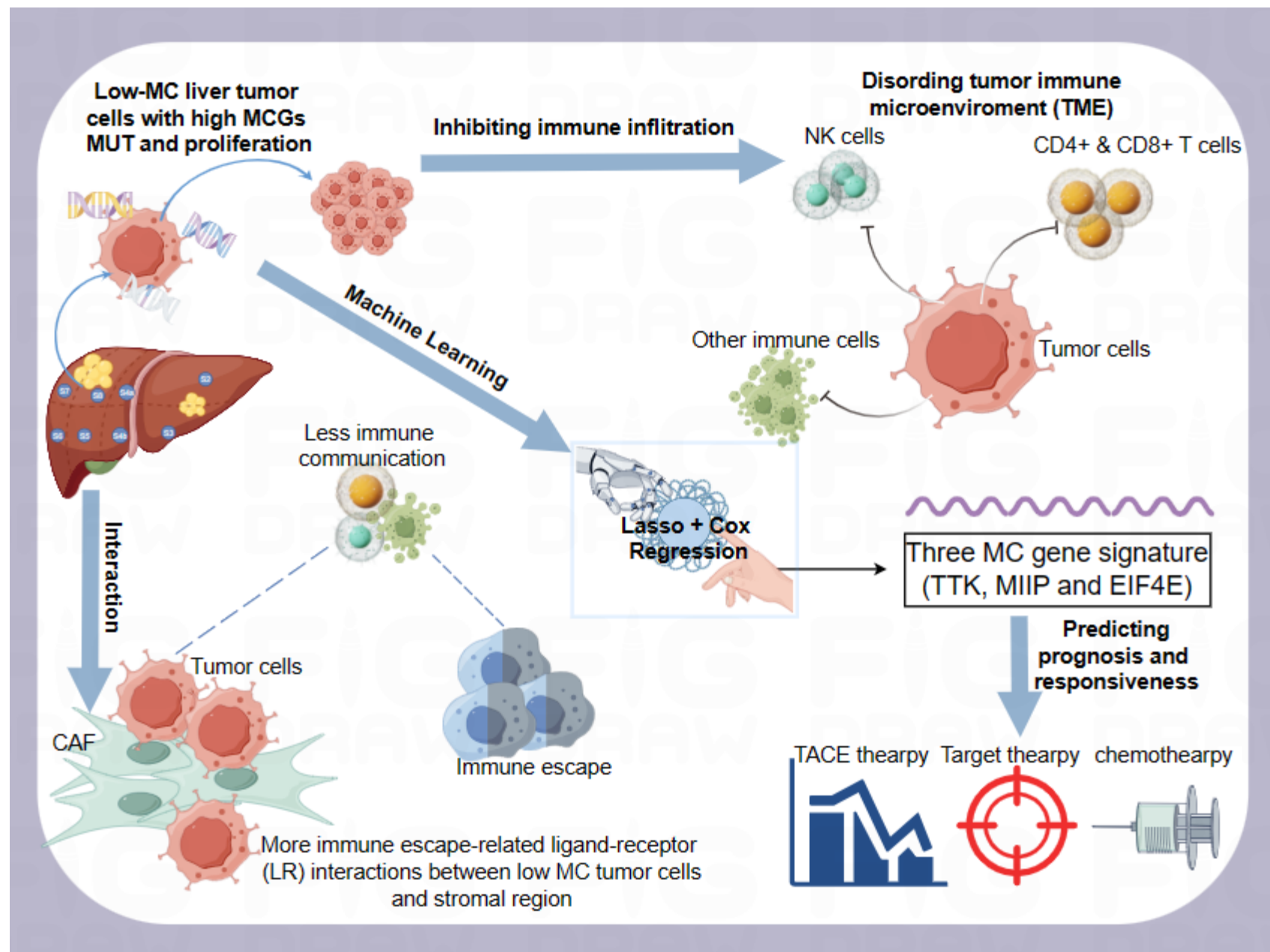

Supplement: Supplementary file 1 [file DataSheet_1.pdf]

*Bulk RNAseq*

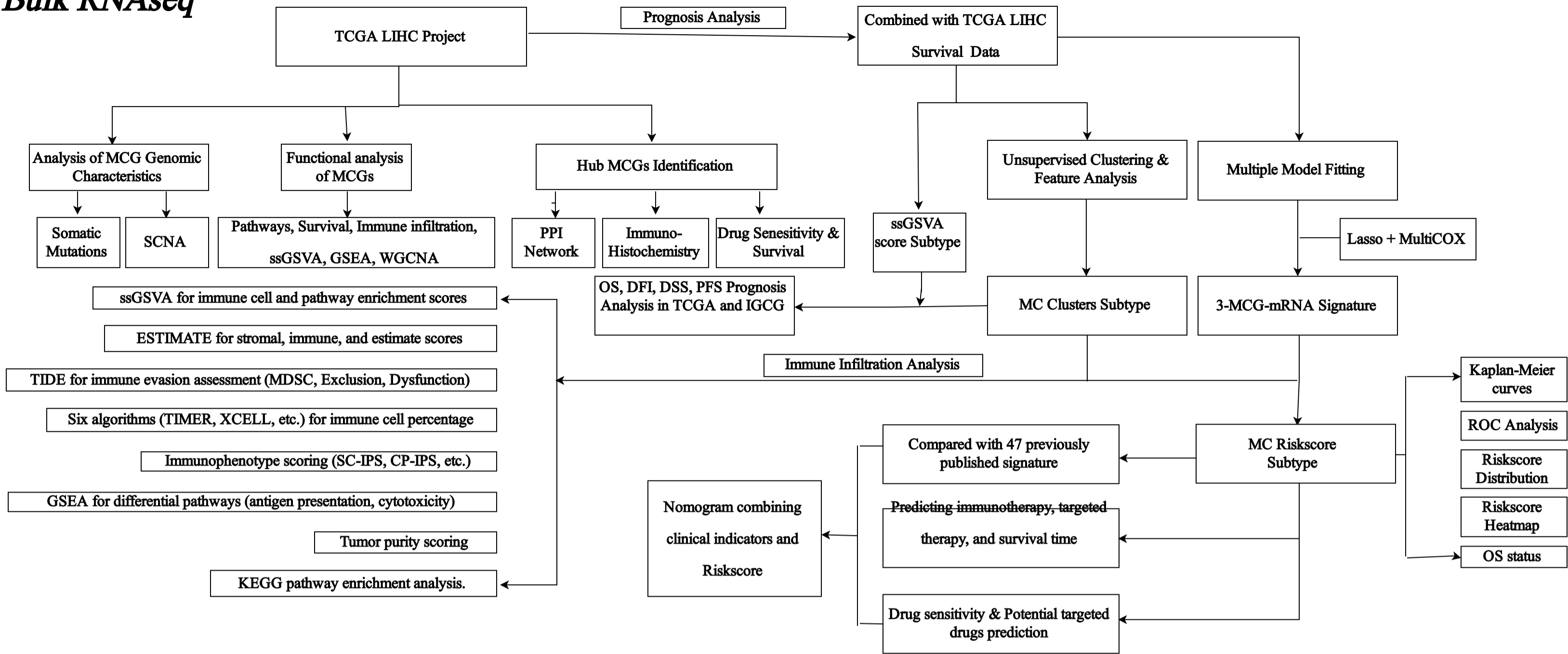

*Single-cell RNA transcriptomics*

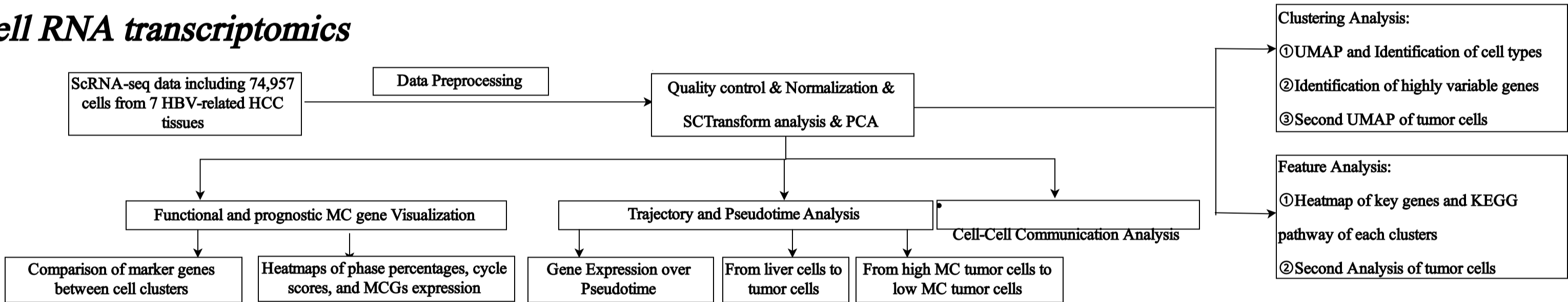

*Spatial transcriptomics*

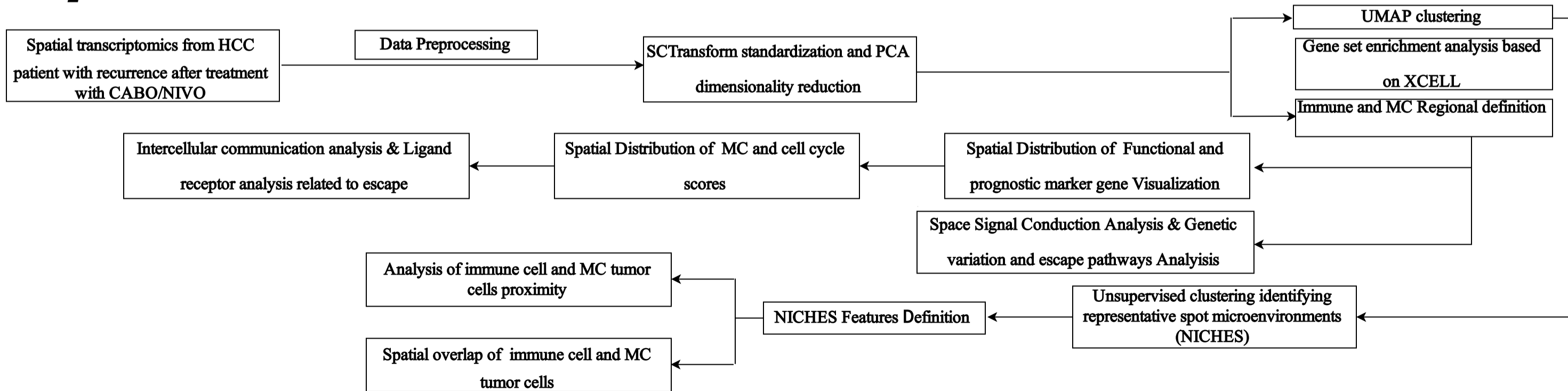

Supplement: Supplementary Figure 1 — Study design and analytical workflow. [file Presentation_1.zip › Image 1.pdf]

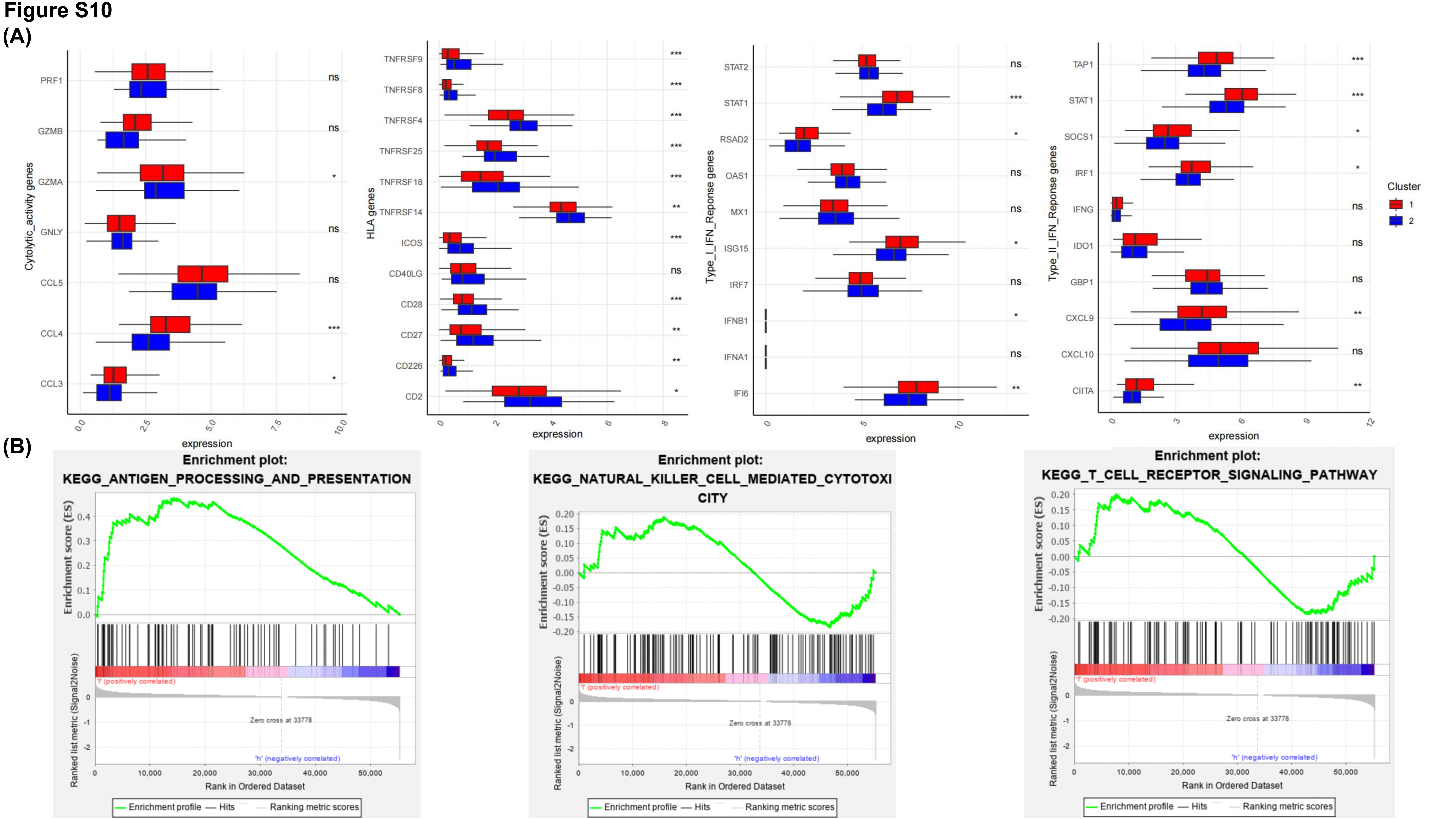

Supplement: Supplementary Figure 1 — Study design and analytical workflow. [file Presentation_1.zip › Image 10.jpg]

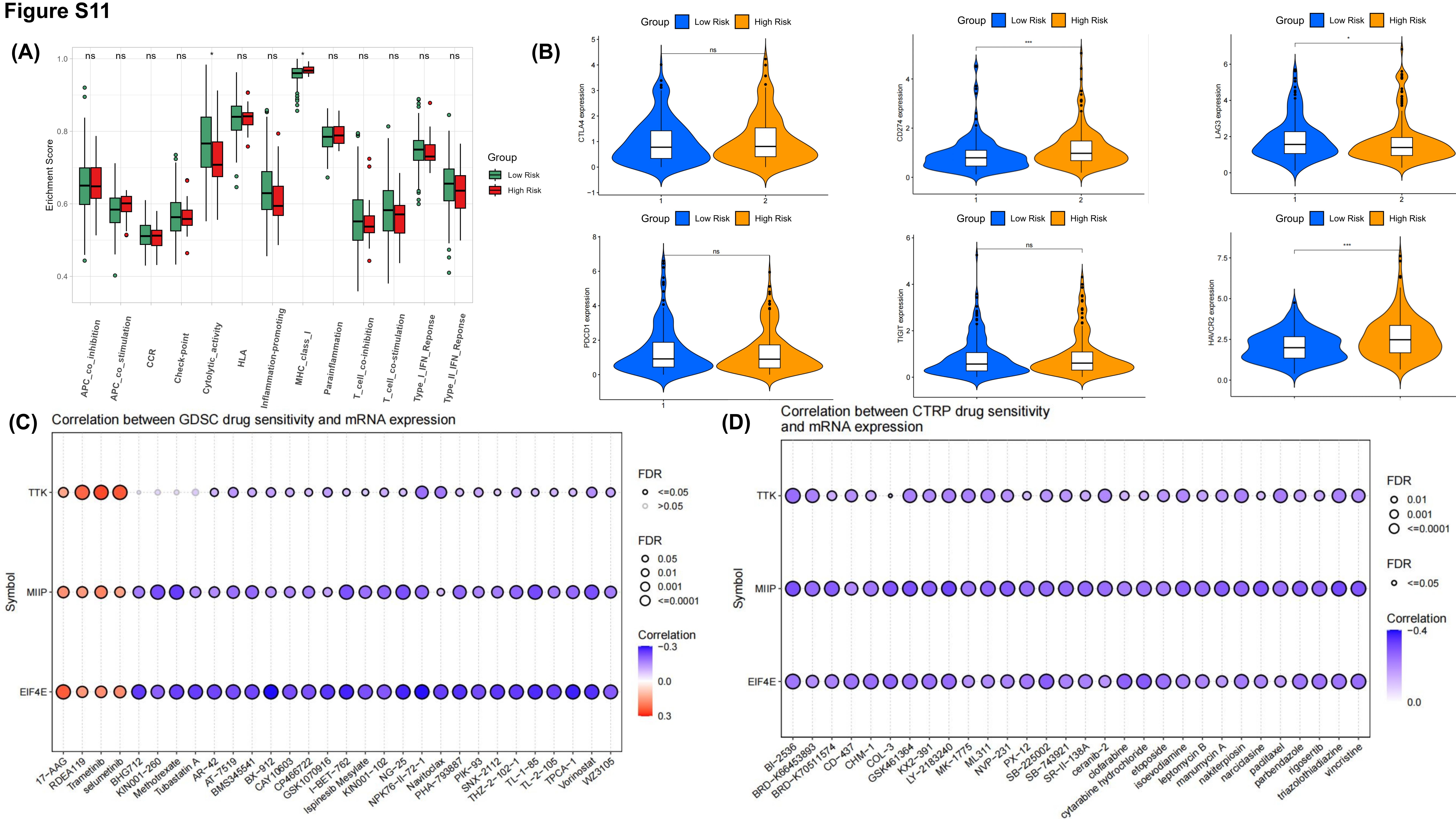

Supplement: Supplementary Figure 1 — Study design and analytical workflow. [file Presentation_1.zip › Image 11.jpg]

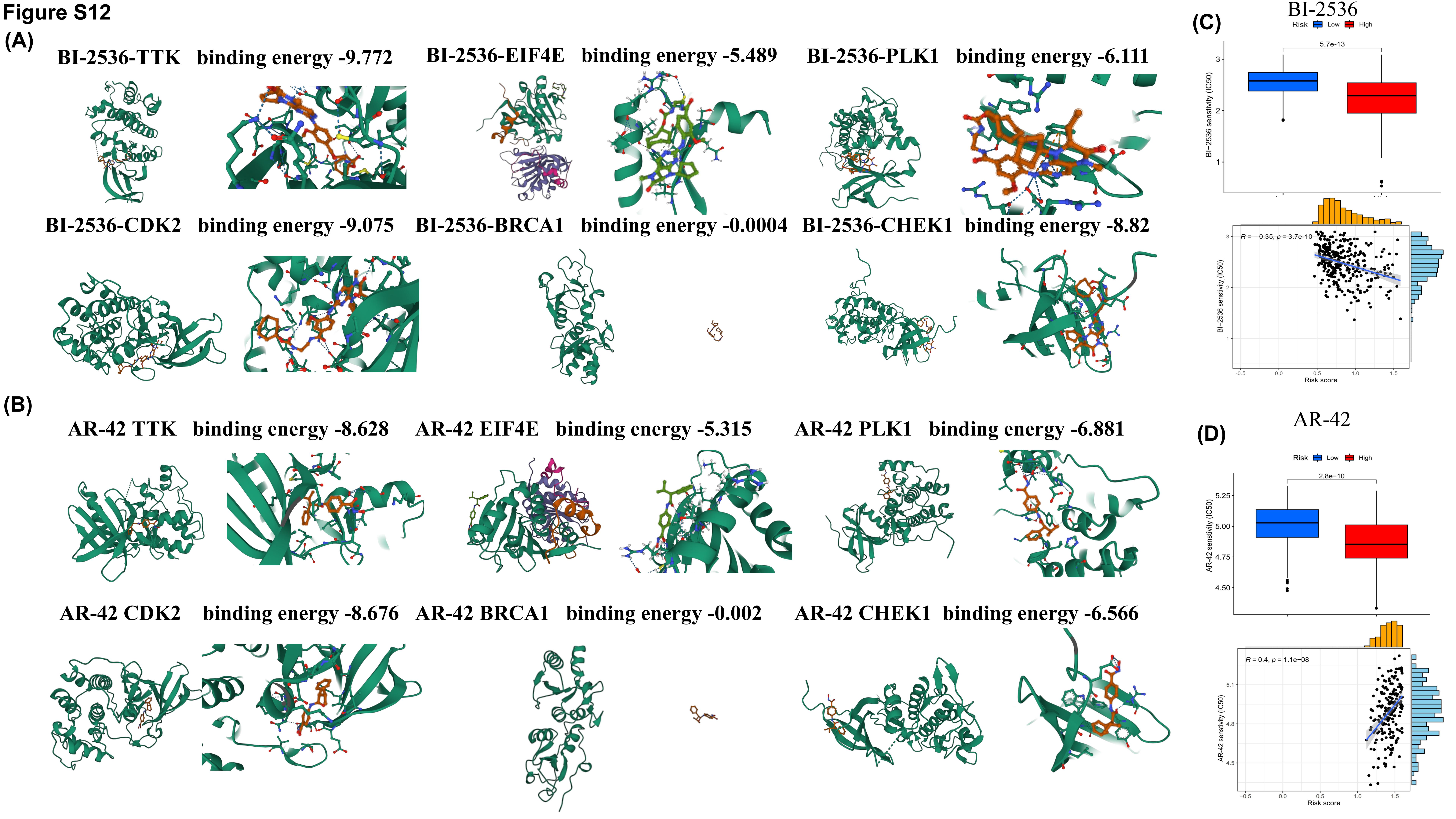

Supplement: Supplementary Figure 1 — Study design and analytical workflow. [file Presentation_1.zip › Image 12.jpg]

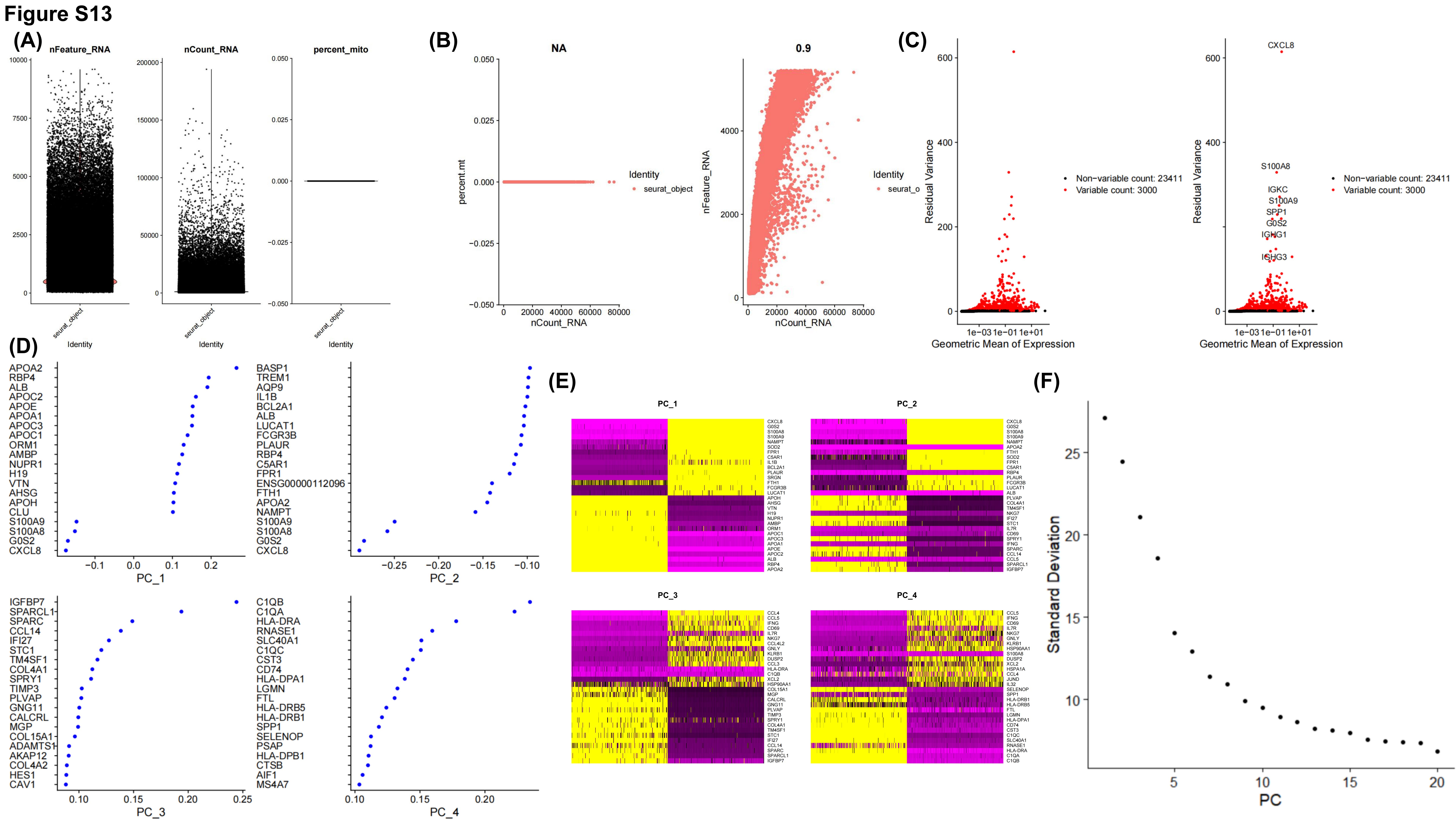

Supplement: Supplementary Figure 1 — Study design and analytical workflow. [file Presentation_1.zip › Image 13.jpg]

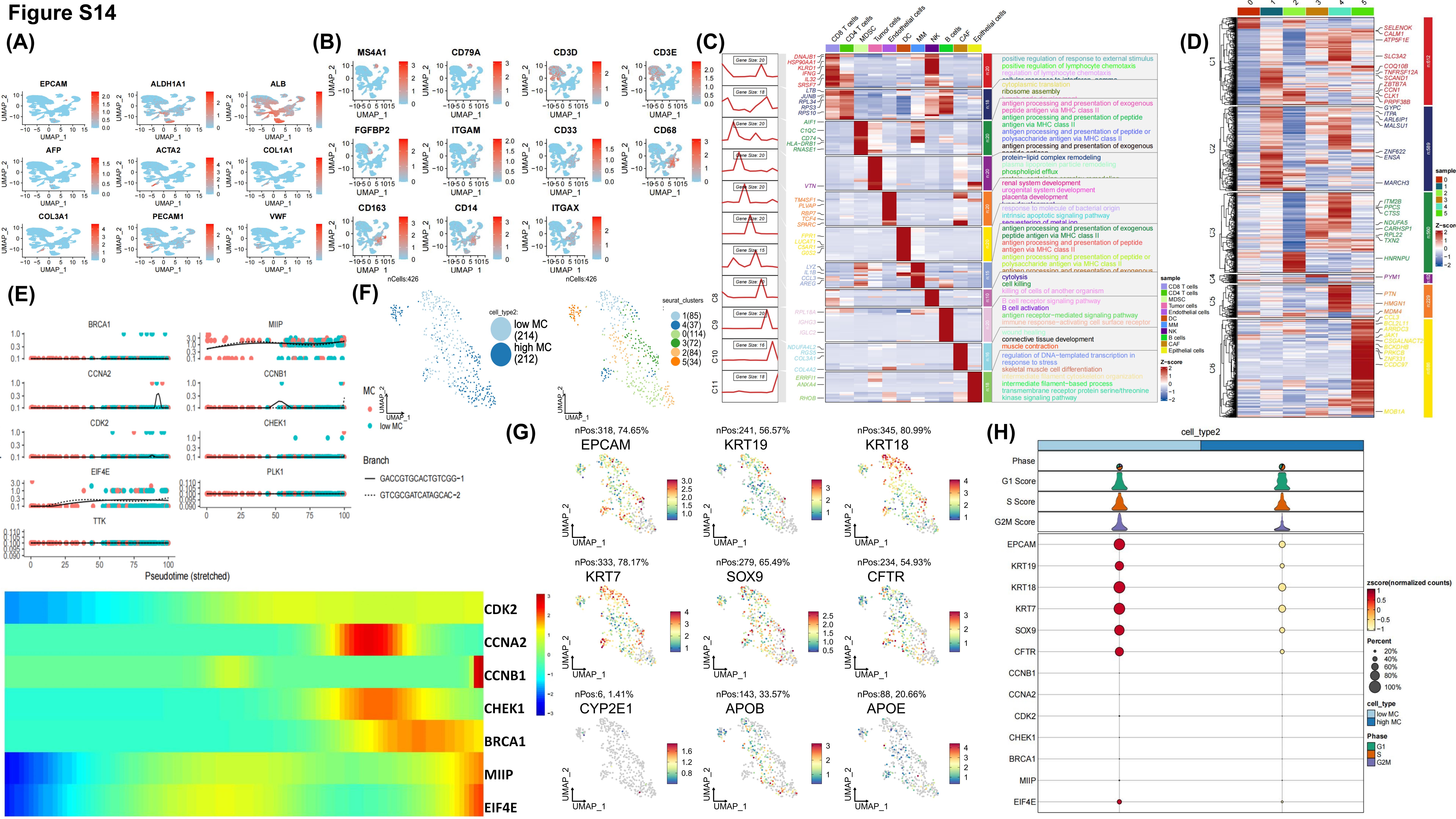

Supplement: Supplementary Figure 1 — Study design and analytical workflow. [file Presentation_1.zip › Image 14.jpg]

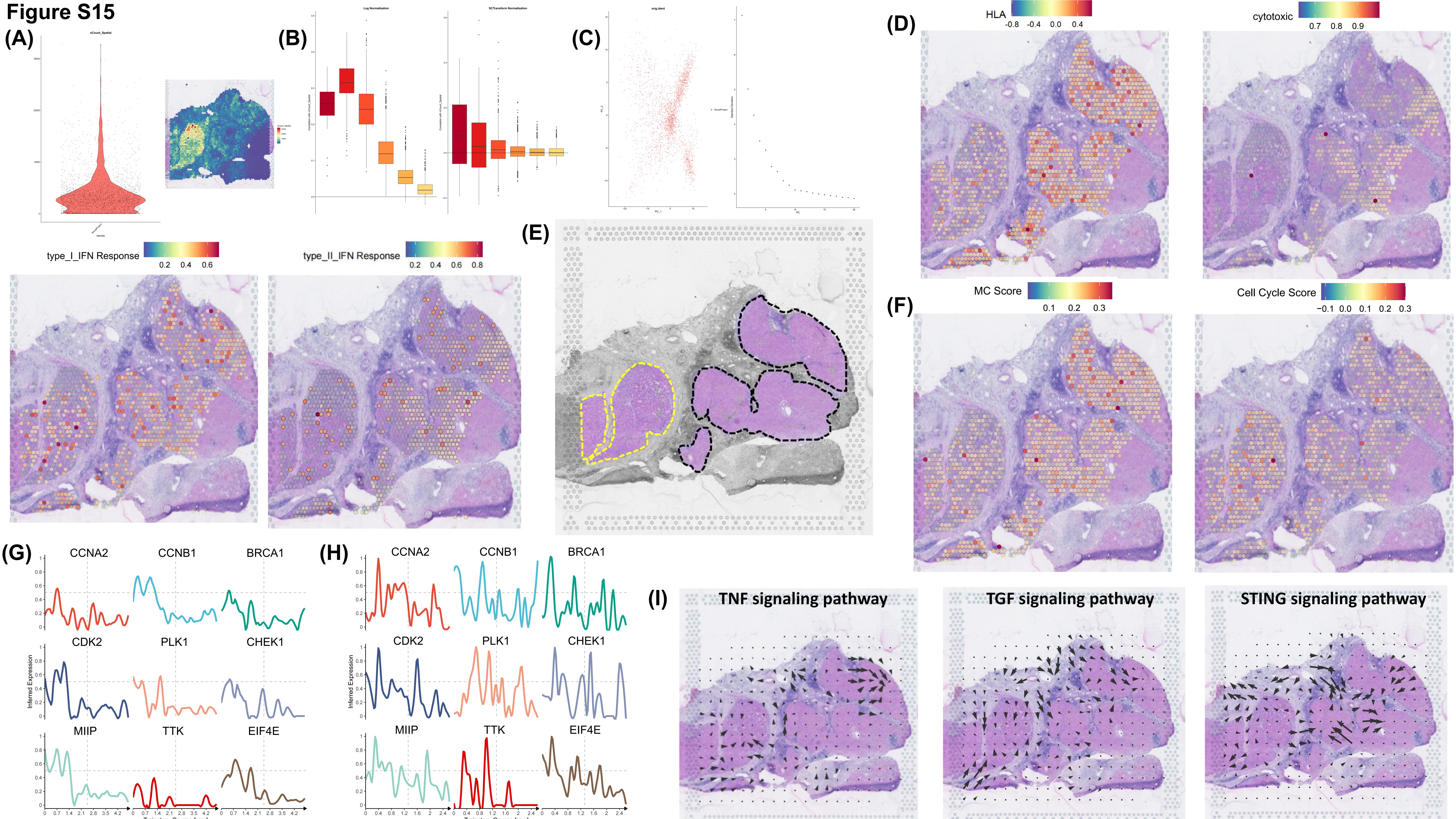

Supplement: Supplementary Figure 1 — Study design and analytical workflow. [file Presentation_1.zip › Image 15.jpg]

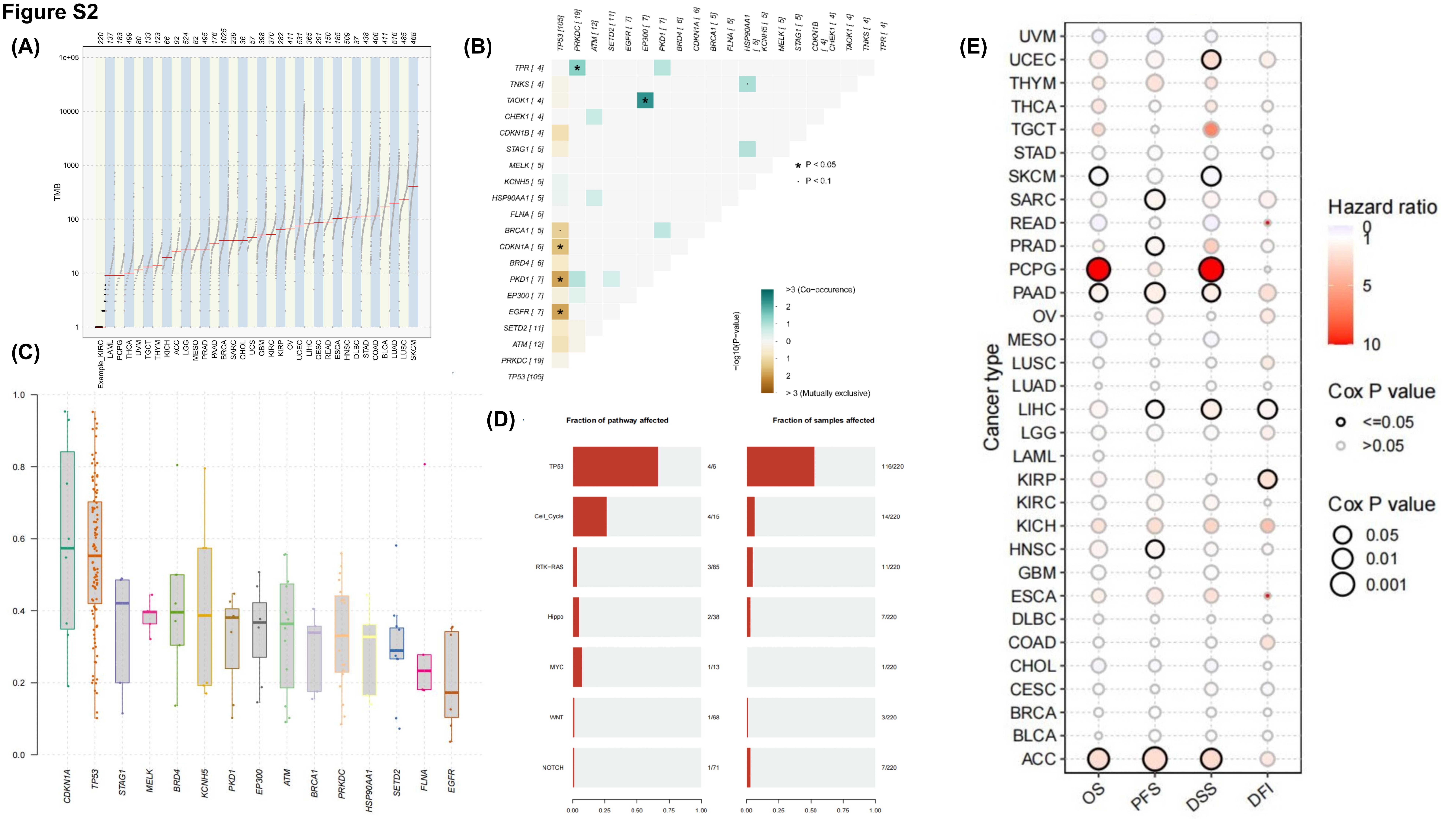

Supplement: Supplementary Figure 1 — Study design and analytical workflow. [file Presentation_1.zip › Image 2.jpg]

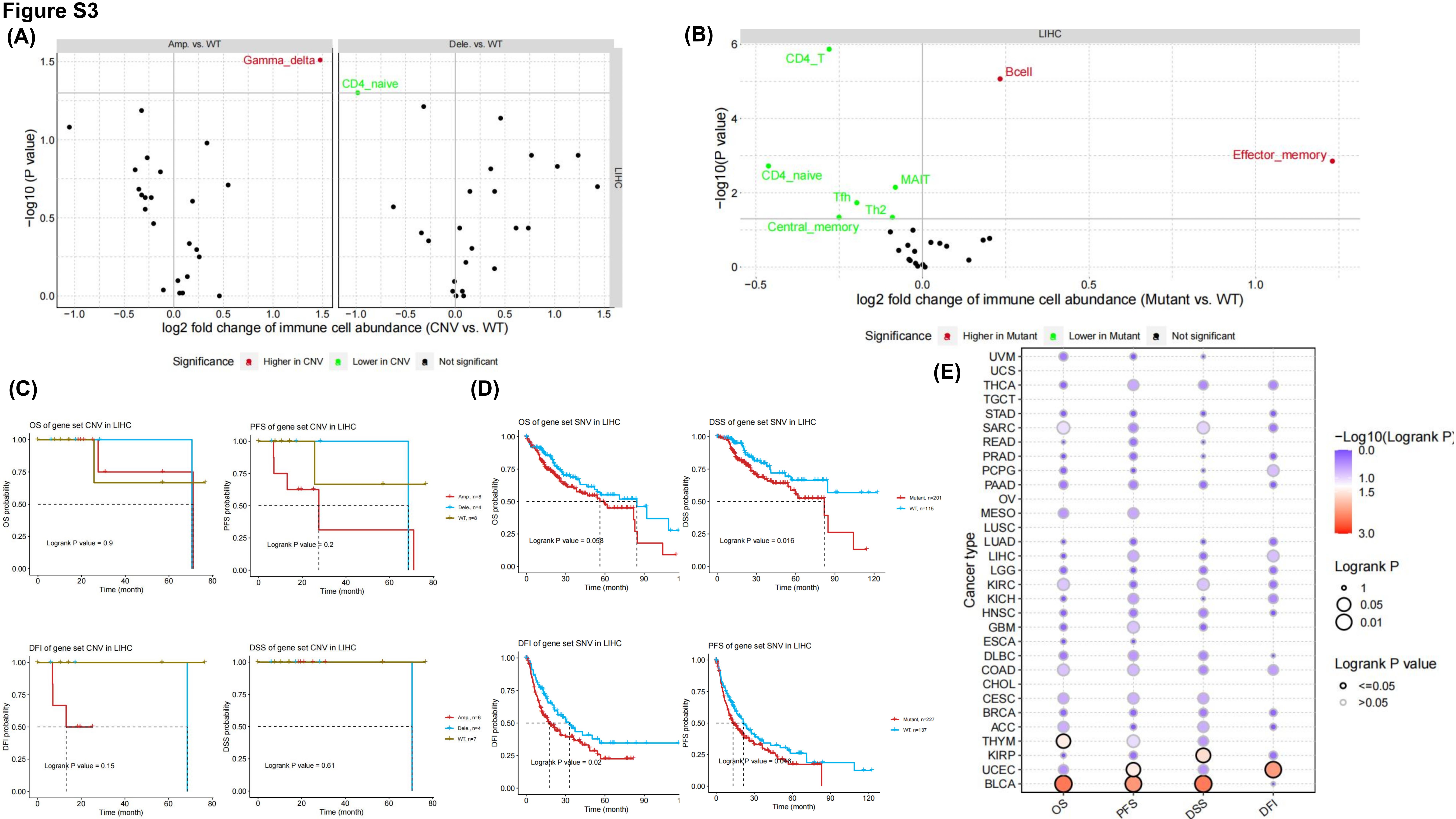

Supplement: Supplementary Figure 1 — Study design and analytical workflow. [file Presentation_1.zip › Image 3.jpg]

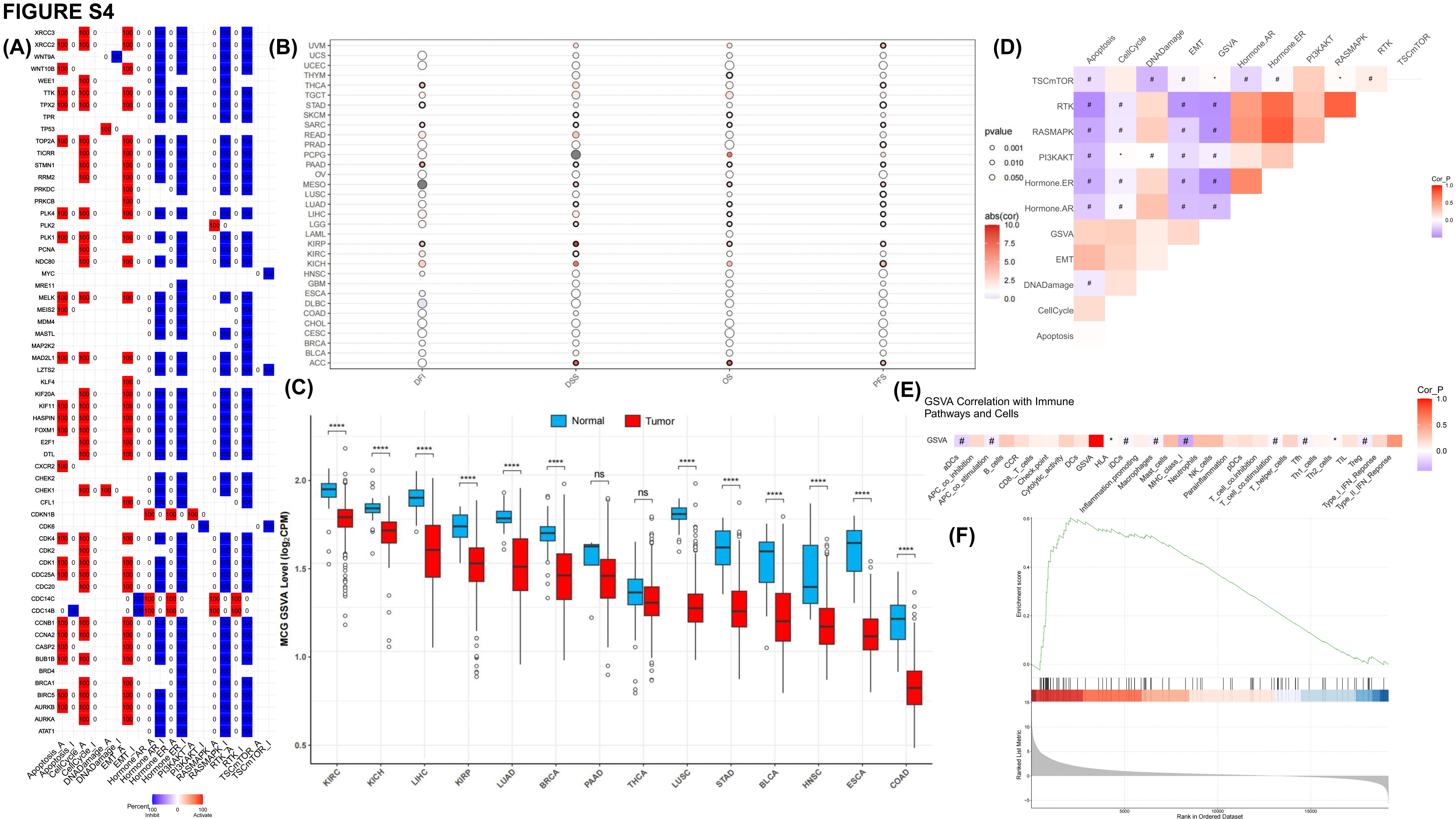

Supplement: Supplementary Figure 1 — Study design and analytical workflow. [file Presentation_1.zip › Image 4.jpg]

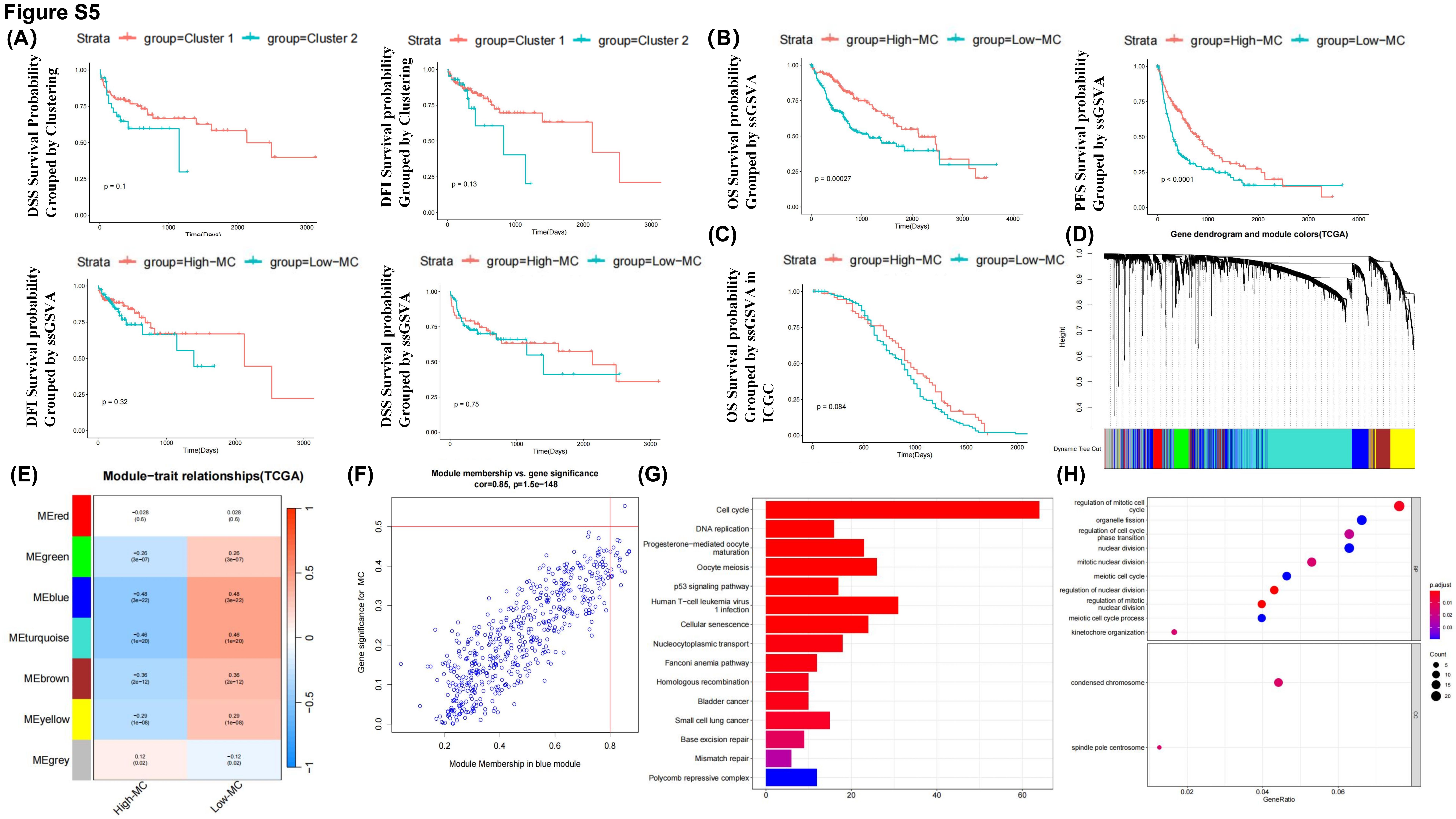

Supplement: Supplementary Figure 1 — Study design and analytical workflow. [file Presentation_1.zip › Image 5.jpg]

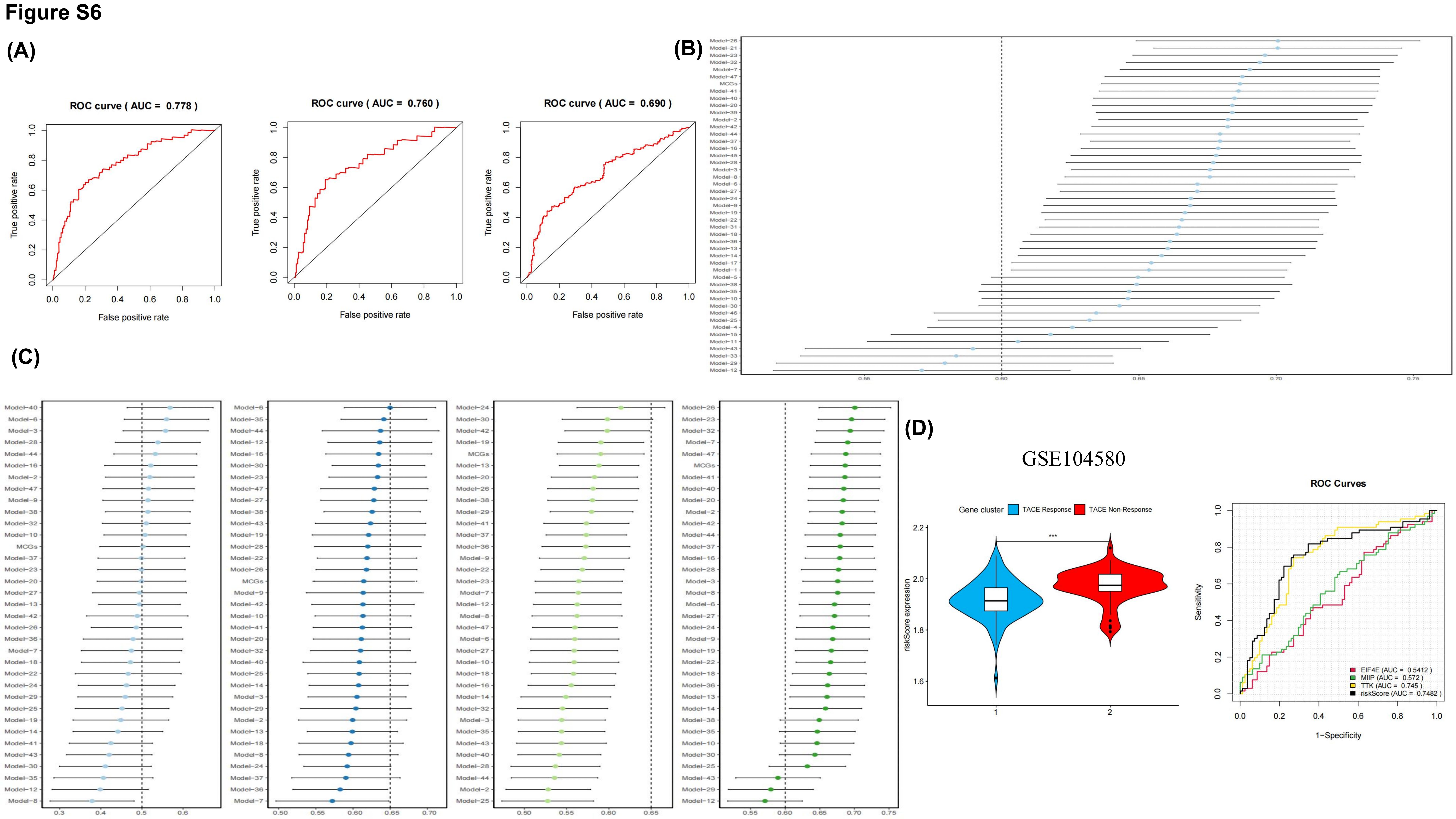

Supplement: Supplementary Figure 1 — Study design and analytical workflow. [file Presentation_1.zip › Image 6.jpg]

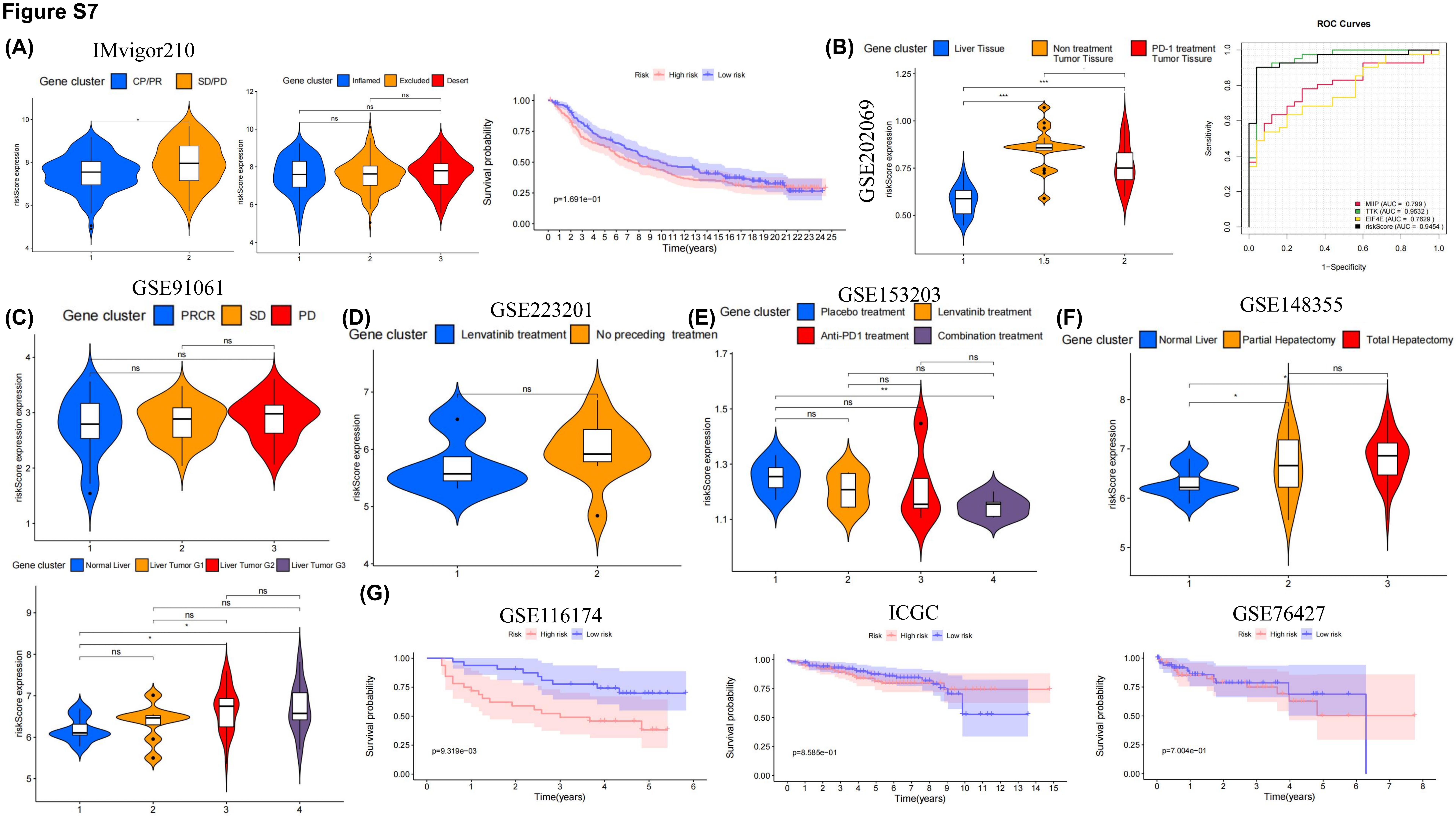

Supplement: Supplementary Figure 1 — Study design and analytical workflow. [file Presentation_1.zip › Image 7.jpg]

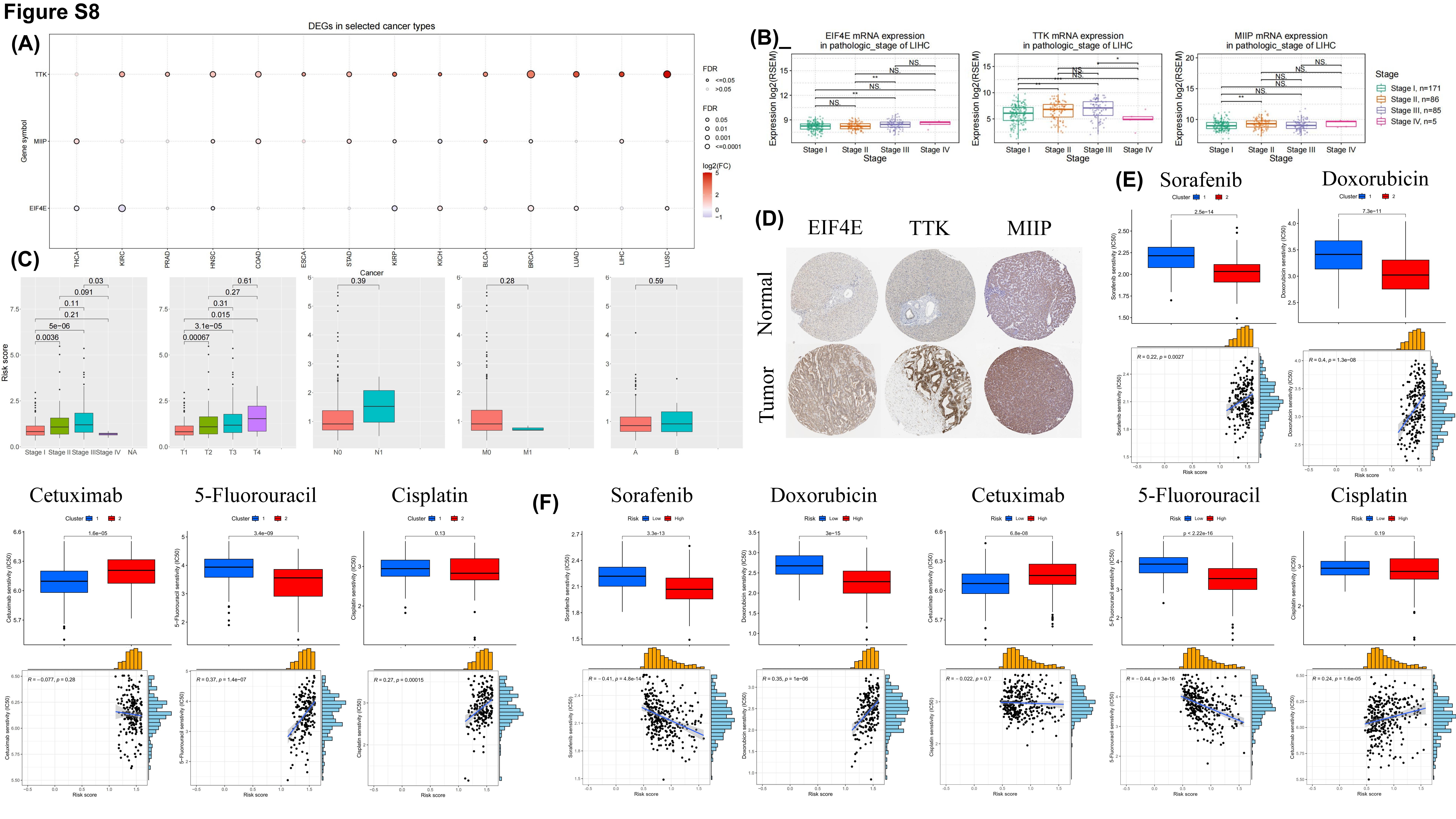

Supplement: Supplementary Figure 1 — Study design and analytical workflow. [file Presentation_1.zip › Image 8.jpg]

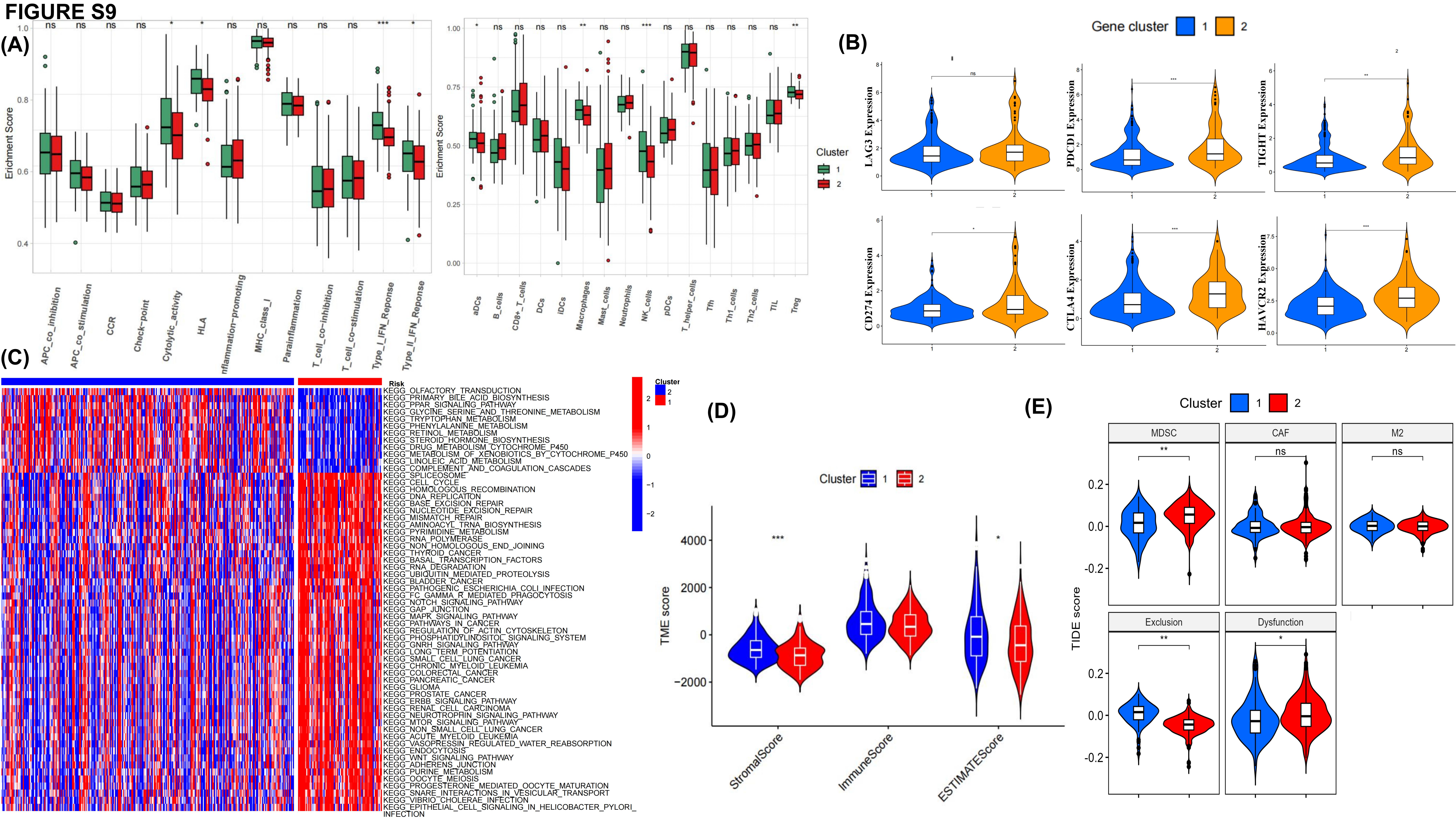

Supplement: Supplementary Figure 1 — Study design and analytical workflow. [file Presentation_1.zip › Image 9.jpg]
