# Supplementary material for: Mitotic catastrophe heterogeneity: implications for prognosis and immunotherapy in hepatocellular carcinoma
Source: Front Immunol. 2024 Jul 1;15:1409448. doi: 10.3389/fimmu.2024.1409448 (PMC11250588; doi:10.3389/fimmu.2024.1409448)
Supplement: Supplementary file 2 [file Table_1.docx]

**Supplementary Table S1: 3 MCGs significantly associated with OS of HCC patients**

| ID | Coef | HR | HR.95L | HR.95H | p-value |
| --- | --- | --- | --- | --- | --- |
| EIF4E | 0.443496678 | 1.558146039 | 1.0462317 | 2.320536721 | 0.029083059 |
| TTK | 0.278545722 | 1.321207012 | 1.093649869 | 1.596112264 | 0.003874714 |
| MIIP | 0.237553637 | 1.268143015 | 0.977120815 | 1.645842235 | 0.074106183 |
