# Supplementary material for: Mitotic catastrophe heterogeneity: implications for prognosis and immunotherapy in hepatocellular carcinoma
Source: Front Immunol. 2024 Jul 1;15:1409448. doi: 10.3389/fimmu.2024.1409448 (PMC11250588; doi:10.3389/fimmu.2024.1409448)
Supplement: Supplementary file 3 [file Table_2.docx]

| **Supplementary Table S2: A total of 47 published signatures were**  **retrieved from the literatures** | | | | | |
| --- | --- | --- | --- | --- | --- |
| **Model** | **PMID** | **Type** | **Author** | **ENSEMBL** | **Coef** |
| Model-1 | 36017840 | mRNA | Li | ENSG00000149089 | 0.0326 |
| Model-1 | 36017840 | mRNA | Li | ENSG00000135829 | 0.1677 |
| Model-1 | 36017840 | mRNA | Li | ENSG00000147697 | 0.0642 |
| Model-1 | 36017840 | mRNA | Li | ENSG00000105928 | 0.1139 |
| Model-1 | 36017840 | mRNA | Li | ENSG00000091106 | 0.0028 |
| Model-1 | 36017840 | mRNA | Li | ENSG00000174885 | -0.1000 |
| Model-1 | 36017840 | mRNA | Li | ENSG00000167207 | 0.0473 |
| Model-1 | 36017840 | mRNA | Li | ENSG00000139218 | 0.0388 |
| Model-1 | 36017840 | mRNA | Li | ENSG00000095970 | 0.0239 |
| Model-2 | 37383726 | mRNA | Xu | ENSG00000111640 | 0.1481 |
| Model-2 | 37383726 | mRNA | Xu | ENSG00000168615 | 0.1581 |
| Model-2 | 37383726 | mRNA | Xu | ENSG00000077463 | 0.1247 |
| Model-2 | 37383726 | mRNA | Xu | ENSG00000131981 | 0.0666 |
| Model-2 | 37383726 | mRNA | Xu | ENSG00000073754 | -0.0144 |
| Model-2 | 37383726 | mRNA | Xu | ENSG00000137033 | -0.0985 |
| Model-3 | 37034303 | mRNA | Dai | ENSG00000214160 | 0.0590 |
| Model-3 | 37034303 | mRNA | Dai | ENSG00000106392 | 0.1770 |
| Model-3 | 37034303 | mRNA | Dai | ENSG00000172269 | 0.1540 |
| Model-3 | 37034303 | mRNA | Dai | ENSG00000087250 | 0.1860 |
| Model-3 | 37034303 | mRNA | Dai | ENSG00000009830 | 0.2130 |
| Model-3 | 37034303 | mRNA | Dai | ENSG00000163902 | 0.1030 |
| Model-3 | 37034303 | mRNA | Dai | ENSG00000136840 | 0.2640 |
| Model-3 | 37034303 | mRNA | Dai | ENSG00000134910 | 0.0850 |
| Model-4 | 37149620 | mRNA | Chen | ENSG00000030110 | 0.0280 |
| Model-4 | 37149620 | mRNA | Chen | ENSG00000105928 | 0.1240 |
| Model-4 | 37149620 | mRNA | Chen | ENSG00000174885 | -0.1350 |
| Model-4 | 37149620 | mRNA | Chen | ENSG00000167207 | 0.4470 |
| Model-5 | 10260314 | mRNA | Xiong | ENSG00000135341 | 0.3156 |
| Model-5 | 10260314 | mRNA | Xiong | ENSG00000179526 | 0.2255 |
| Model-5 | 10260314 | mRNA | Xiong | ENSG00000170312 | 0.2624 |
| Model-6 | 37051238 | mRNA | Sun | ENSG00000172270 | 0.1756 |
| Model-6 | 37051238 | mRNA | Sun | ENSG00000112984 | 0.2711 |
| Model-6 | 37051238 | mRNA | Sun | ENSG00000101236 | 0.3953 |
| Model-7 | 36726530 | mRNA | Zheng | ENSG00000087250 | 0.0966 |
| Model-7 | 36726530 | mRNA | Zheng | ENSG00000198431 | 0.0126 |
| Model-7 | 36726530 | mRNA | Zheng | ENSG00000117632 | 0.0257 |
| Model-8 | 37316840 | mRNA | Sun | ENSG00000069482 | 0.3590 |
| Model-8 | 37316840 | mRNA | Sun | ENSG00000169297 | 0.4540 |
| Model-8 | 37316840 | mRNA | Sun | ENSG00000186868 | 0.4950 |
| Model-8 | 37316840 | mRNA | Sun | ENSG00000183625 | 0.6410 |
| Model-8 | 37316840 | mRNA | Sun | ENSG00000112164 | 0.3060 |
| Model-8 | 37316840 | mRNA | Sun | ENSG00000089685 | 0.4280 |
| Model-8 | 37316840 | mRNA | Sun | ENSG00000127743 | 2.1000 |
| Model-9 | 37324188 | mRNA | Song | ENSG00000110047 | 0.0389 |
| Model-9 | 37324188 | mRNA | Song | ENSG00000136238 | 0.2019 |
| Model-9 | 37324188 | mRNA | Song | ENSG00000116560 | 0.3577 |
| Model-9 | 37324188 | mRNA | Song | ENSG00000153071 | 0.0352 |
| Model-9 | 37324188 | mRNA | Song | ENSG00000090621 | 0.0613 |
| Model-10 | 35841206 | mRNA | Long | ENSG00000151012 | 0.2653 |
| Model-10 | 35841206 | mRNA | Long | ENSG00000105281 | 0.2343 |
| Model-10 | 35841206 | mRNA | Long | ENSG00000023909 | 0.2126 |
| Model-10 | 35841206 | mRNA | Long | ENSG00000130066 | -0.2949 |
| Model-11 | 35419294 | mRNA | Zhou | ENSG00000140534 | -0.1666 |
| Model-11 | 35419294 | mRNA | Zhou | ENSG00000040275 | -0.1195 |
| Model-11 | 35419294 | mRNA | Zhou | ENSG00000129810 | 0.0722 |
| Model-11 | 35419294 | mRNA | Zhou | ENSG00000123485 | 0.0209 |
| Model-11 | 35419294 | mRNA | Zhou | ENSG00000115163 | 0.0889 |
| Model-11 | 35419294 | mRNA | Zhou | ENSG00000101003 | 0.0595 |
| Model-11 | 35419294 | mRNA | Zhou | ENSG00000106462 | 0.0374 |
| Model-11 | 35419294 | mRNA | Zhou | ENSG00000160469 | -0.1731 |
| Model-11 | 35419294 | mRNA | Zhou | ENSG00000143228 | 0.0677 |
| Model-11 | 35419294 | mRNA | Zhou | ENSG00000166851 | 0.0001 |
| Model-11 | 35419294 | mRNA | Zhou | ENSG00000072571 | 0.1552 |
| Model-11 | 35419294 | mRNA | Zhou | ENSG00000007968 | -1.3701 |
| Model-11 | 35419294 | mRNA | Zhou | ENSG00000134690 | 0.1812 |
| Model-12 | 36911742 | mRNA | Li | ENSG00000047936 | -0.5071 |
| Model-12 | 36911742 | mRNA | Li | ENSG00000065526 | -0.5189 |
| Model-12 | 36911742 | mRNA | Li | ENSG00000196090 | -0.3485 |
| Model-13 | 37006257 | mRNA | Chi | ENSG00000206503 | -0.0712 |
| Model-13 | 37006257 | mRNA | Chi | ENSG00000078596 | -0.0878 |
| Model-13 | 37006257 | mRNA | Chi | ENSG00000143851 | -0.1846 |
| Model-13 | 37006257 | mRNA | Chi | ENSG00000147168 | 0.1181 |
| Model-13 | 37006257 | mRNA | Chi | ENSG00000227507 | -0.1403 |
| Model-13 | 37006257 | mRNA | Chi | ENSG00000186827 | 0.2926 |
| Model-13 | 37006257 | mRNA | Chi | ENSG00000186891 | -0.0811 |
| Model-13 | 37006257 | mRNA | Chi | ENSG00000034510 | 0.0145 |
| Model-13 | 37006257 | mRNA | Chi | ENSG00000175463 | -0.1721 |
| Model-13 | 37006257 | mRNA | Chi | ENSG00000130429 | 0.1368 |
| Model-13 | 37006257 | mRNA | Chi | ENSG00000109861 | 0.2523 |
| Model-14 | 36750831 | mRNA | Chen | ENSG00000105707 | -0.5910 |
| Model-14 | 36750831 | mRNA | Chen | ENSG00000189050 | 0.5342 |
| Model-14 | 36750831 | mRNA | Chen | ENSG00000160209 | 0.7707 |
| Model-14 | 36750831 | mRNA | Chen | ENSG00000155287 | -1.0743 |
| Model-14 | 36750831 | mRNA | Chen | ENSG00000163815 | -0.6489 |
| Model-15 | 32631357 | mRNA | Du | ENSG00000237289 | -0.1579 |
| Model-15 | 32631357 | mRNA | Du | ENSG00000141934 | -0.0607 |
| Model-15 | 32631357 | mRNA | Du | ENSG00000144366 | 0.1118 |
| Model-15 | 32631357 | mRNA | Du | ENSG00000109674 | 0.1148 |
| Model-15 | 32631357 | mRNA | Du | ENSG00000248144 | -0.1013 |
| Model-15 | 32631357 | mRNA | Du | ENSG00000066923 | -0.1999 |
| Model-15 | 32631357 | mRNA | Du | ENSG00000078596 | -0.3719 |
| Model-16 | 34557495 | mRNA | Zhao | ENSG00000149541 | 0.0068 |
| Model-16 | 34557495 | mRNA | Zhao | ENSG00000117411 | 0.0123 |
| Model-16 | 34557495 | mRNA | Zhao | ENSG00000100243 | 0.0001 |
| Model-16 | 34557495 | mRNA | Zhao | ENSG00000113552 | 0.0072 |
| Model-16 | 34557495 | mRNA | Zhao | ENSG00000125166 | -0.0006 |
| Model-16 | 34557495 | mRNA | Zhao | ENSG00000049860 | 0.0000 |
| Model-16 | 34557495 | mRNA | Zhao | ENSG00000152952 | 0.0181 |
| Model-16 | 34557495 | mRNA | Zhao | ENSG00000086475 | 0.0601 |
| Model-17 | 35047554 | mRNA | Zhang | ENSG00000030110 | 0.0749 |
| Model-17 | 35047554 | mRNA | Zhang | ENSG00000101421 | 0.1449 |
| Model-17 | 35047554 | mRNA | Zhang | ENSG00000147697 | 0.1517 |
| Model-17 | 35047554 | mRNA | Zhang | ENSG00000174885 | -0.3092 |
| Model-17 | 35047554 | mRNA | Zhang | ENSG00000167207 | 0.2718 |
| Model-17 | 35047554 | mRNA | Zhang | ENSG00000124181 | 0.0098 |
| Model-17 | 35047554 | mRNA | Zhang | ENSG00000139218 | 0.2083 |
| Model-18 | 35193632 | mRNA | Hu | ENSG00000117586 | 0.2558 |
| Model-18 | 35193632 | mRNA | Hu | ENSG00000167664 | -0.2900 |
| Model-18 | 35193632 | mRNA | Hu | ENSG00000186827 | 0.1338 |
| Model-18 | 35193632 | mRNA | Hu | ENSG00000164761 | 0.2201 |
| Model-18 | 35193632 | mRNA | Hu | ENSG00000141655 | 0.4021 |
| Model-18 | 35193632 | mRNA | Hu | ENSG00000102245 | -0.7810 |
| Model-19 | 33712026 | mRNA | Zhu | ENSG00000249915 | 0.0495 |
| Model-19 | 33712026 | mRNA | Zhu | ENSG00000070814 | 0.1605 |
| Model-19 | 33712026 | mRNA | Zhu | ENSG00000130726 | -0.0070 |
| Model-19 | 33712026 | mRNA | Zhu | ENSG00000106462 | 0.1388 |
| Model-19 | 33712026 | mRNA | Zhu | ENSG00000101447 | 0.0485 |
| Model-20 | 33568167 | mRNA | Peng | ENSG00000143546 | 0.0766 |
| Model-20 | 33568167 | mRNA | Peng | ENSG00000089685 | 0.0299 |
| Model-20 | 33568167 | mRNA | Peng | ENSG00000116161 | 0.1778 |
| Model-20 | 33568167 | mRNA | Peng | ENSG00000169297 | 0.0960 |
| Model-20 | 33568167 | mRNA | Peng | ENSG00000164520 | 0.3173 |
| Model-20 | 33568167 | mRNA | Peng | ENSG00000133710 | -0.1412 |
| Model-20 | 33568167 | mRNA | Peng | ENSG00000118785 | 0.0800 |
| Model-21 | 34150630 | mRNA | Yuan | ENSG00000160211 | 0.2098 |
| Model-21 | 34150630 | mRNA | Yuan | ENSG00000227471 | 0.1237 |
| Model-21 | 34150630 | mRNA | Yuan | ENSG00000072571 | 0.2305 |
| Model-21 | 34150630 | mRNA | Yuan | ENSG00000114646 | 0.2112 |
| Model-21 | 34150630 | mRNA | Yuan | ENSG00000119915 | 0.2719 |
| Model-21 | 34150630 | mRNA | Yuan | ENSG00000170231 | 0.0373 |
| Model-22 | 32913470 | mRNA | Xie | ENSG00000163931 | 0.3450 |
| Model-22 | 32913470 | mRNA | Xie | ENSG00000078699 | 0.2130 |
| Model-22 | 32913470 | mRNA | Xie | ENSG00000162073 | 0.1320 |
| Model-22 | 32913470 | mRNA | Xie | ENSG00000160298 | 0.1150 |
| Model-22 | 32913470 | mRNA | Xie | ENSG00000155158 | -0.0150 |
| Model-22 | 32913470 | mRNA | Xie | ENSG00000198523 | -0.0430 |
| Model-22 | 32913470 | mRNA | Xie | ENSG00000169271 | -0.0770 |
| Model-23 | 34721731 | mRNA | Hui | ENSG00000112984 | 0.0472 |
| Model-23 | 34721731 | mRNA | Hui | ENSG00000134690 | 0.0632 |
| Model-23 | 34721731 | mRNA | Hui | ENSG00000182481 | 0.0551 |
| Model-23 | 34721731 | mRNA | Hui | ENSG00000160211 | 0.0910 |
| Model-23 | 34721731 | mRNA | Hui | ENSG00000104419 | 0.0337 |
| Model-23 | 34721731 | mRNA | Hui | ENSG00000198758 | 0.0066 |
| Model-24 | 33725827 | mRNA | Liu | ENSG00000171388 | 0.1640 |
| Model-24 | 33725827 | mRNA | Liu | ENSG00000135446 | 0.2540 |
| Model-24 | 33725827 | mRNA | Liu | ENSG00000081041 | -0.0790 |
| Model-24 | 33725827 | mRNA | Liu | ENSG00000091831 | -0.1990 |
| Model-24 | 33725827 | mRNA | Liu | ENSG00000136689 | -0.1990 |
| Model-24 | 33725827 | mRNA | Liu | ENSG00000175166 | 0.1660 |
| Model-24 | 33725827 | mRNA | Liu | ENSG00000001617 | 0.2460 |
| Model-24 | 33725827 | mRNA | Liu | ENSG00000118785 | 0.0750 |
| Model-25 | 33778129 | mRNA | Xu | ENSG00000196611 | 0.1488 |
| Model-25 | 33778129 | mRNA | Xu | ENSG00000134240 | -0.0393 |
| Model-25 | 33778129 | mRNA | Xu | ENSG00000083807 | -0.0479 |
| Model-26 | 33437359 | mRNA | Jiang | ENSG00000100823 | 0.0050 |
| Model-26 | 33437359 | mRNA | Jiang | ENSG00000175054 | 0.0002 |
| Model-26 | 33437359 | mRNA | Jiang | ENSG00000064601 | 0.0030 |
| Model-26 | 33437359 | mRNA | Jiang | ENSG00000101152 | 0.0010 |
| Model-26 | 33437359 | mRNA | Jiang | ENSG00000074800 | 0.0002 |
| Model-26 | 33437359 | mRNA | Jiang | ENSG00000130427 | 0.0030 |
| Model-26 | 33437359 | mRNA | Jiang | ENSG00000100292 | 0.0010 |
| Model-26 | 33437359 | mRNA | Jiang | ENSG00000134333 | 0.0040 |
| Model-26 | 33437359 | mRNA | Jiang | ENSG00000104419 | 0.0040 |
| Model-26 | 33437359 | mRNA | Jiang | ENSG00000179094 | -0.0060 |
| Model-27 | 34336648 | mRNA | Su | ENSG00000087586 | 0.0996 |
| Model-27 | 34336648 | mRNA | Su | ENSG00000126838 | -0.1421 |
| Model-27 | 34336648 | mRNA | Su | ENSG00000161800 | 0.3809 |
| Model-27 | 34336648 | mRNA | Su | ENSG00000172497 | -0.0742 |
| Model-27 | 34336648 | mRNA | Su | ENSG00000213398 | -0.1438 |
| Model-28 | 34899826 | mRNA | Huang | ENSG00000130427 | 0.0284 |
| Model-28 | 34899826 | mRNA | Huang | ENSG00000089685 | 0.0248 |
| Model-28 | 34899826 | mRNA | Huang | ENSG00000118785 | 0.0002 |
| Model-29 | 33553243 | mRNA | Zhao | ENSG00000138798 | 0.3842 |
| Model-29 | 33553243 | mRNA | Zhao | ENSG00000126934 | 0.0128 |
| Model-29 | 33553243 | mRNA | Zhao | ENSG00000213281 | 0.0637 |
| Model-29 | 33553243 | mRNA | Zhao | ENSG00000171444 | -0.2675 |
| Model-30 | 35836524 | mRNA | He | ENSG00000064012 | 0.2250 |
| Model-30 | 35836524 | mRNA | He | ENSG00000147697 | 0.3050 |
| Model-30 | 35836524 | mRNA | He | ENSG00000174885 | -0.4790 |
| Model-30 | 35836524 | mRNA | He | ENSG00000167207 | 0.5610 |
| Model-30 | 35836524 | mRNA | He | ENSG00000124181 | 0.1800 |
| Model-31 | 33014055 | mRNA | Mo | ENSG00000120705 | 0.0340 |
| Model-31 | 33014055 | mRNA | Mo | ENSG00000104687 | 0.0067 |
| Model-31 | 33014055 | mRNA | Mo | ENSG00000005020 | 0.0256 |
| Model-31 | 33014055 | mRNA | Mo | ENSG00000144381 | 0.0018 |
| Model-31 | 33014055 | mRNA | Mo | ENSG00000116161 | 0.0203 |
| Model-31 | 33014055 | mRNA | Mo | ENSG00000198805 | 0.0092 |
| Model-32 | 35560794 | mRNA | Chen | ENSG00000087053 | 0.0276 |
| Model-32 | 35560794 | mRNA | Chen | ENSG00000134690 | 0.1250 |
| Model-32 | 35560794 | mRNA | Chen | ENSG00000105281 | 0.0291 |
| Model-32 | 35560794 | mRNA | Chen | ENSG00000160211 | 0.0577 |
| Model-32 | 35560794 | mRNA | Chen | ENSG00000152952 | 0.0469 |
| Model-32 | 35560794 | mRNA | Chen | ENSG00000196611 | 0.0324 |
| Model-32 | 35560794 | mRNA | Chen | ENSG00000117394 | 0.0385 |
| Model-32 | 35560794 | mRNA | Chen | ENSG00000109511 | -0.0136 |
| Model-32 | 35560794 | mRNA | Chen | ENSG00000163220 | 0.0044 |
| Model-32 | 35560794 | mRNA | Chen | ENSG00000118785 | 0.0050 |
| Model-33 | 34147074 | mRNA | Hong | ENSG00000165556 | -0.2030 |
| Model-33 | 34147074 | mRNA | Hong | ENSG00000005421 | -0.0210 |
| Model-33 | 34147074 | mRNA | Hong | ENSG00000198099 | 0.0940 |
| Model-33 | 34147074 | mRNA | Hong | ENSG00000114113 | 0.2970 |
| Model-33 | 34147074 | mRNA | Hong | ENSG00000213398 | 0.1770 |
| Model-33 | 34147074 | mRNA | Hong | ENSG00000069482 | 0.0370 |
| Model-33 | 34147074 | mRNA | Hong | ENSG00000198670 | 0.1020 |
| Model-33 | 34147074 | mRNA | Hong | ENSG00000137869 | 0.0480 |
| Model-33 | 34147074 | mRNA | Hong | ENSG00000184502 | 0.1020 |
| Model-33 | 34147074 | mRNA | Hong | ENSG00000157005 | 0.0870 |
| Model-33 | 34147074 | mRNA | Hong | ENSG00000242366 | 0.1020 |
| Model-34 | 32198063 | mRNA | Zhang | ENSG00000131153 | 0.2530 |
| Model-34 | 32198063 | mRNA | Zhang | ENSG00000164611 | 0.2860 |
| Model-34 | 32198063 | mRNA | Zhang | ENSG00000122952 | 0.3020 |
| Model-34 | 32198063 | mRNA | Zhang | ENSG00000093009 | 0.8250 |
| Model-34 | 32198063 | mRNA | Zhang | ENSG00000164109 | 1.0340 |
| Model-34 | 32198063 | mRNA | Zhang | ENSG00000156970 | 1.7670 |
| Model-34 | 32198063 | mRNA | Zhang | ENSG00000137807 | 3.0330 |
| Model-34 | 32198063 | mRNA | Zhang | ENSG00000142731 | -3.6190 |
| Model-34 | 32198063 | mRNA | Zhang | ENSG00000104147 | -1.4270 |
| Model-34 | 32198063 | mRNA | Zhang | ENSG00000135862 | -1.3180 |
| Model-34 | 32198063 | mRNA | Zhang | ENSG00000157456 | -0.7100 |
| Model-34 | 32198063 | mRNA | Zhang | ENSG00000143476 | -0.5820 |
| Model-34 | 32198063 | mRNA | Zhang | ENSG00000089685 | 0.1960 |
| Model-34 | 32198063 | mRNA | Zhang | ENSG00000104738 | -0.1860 |
| Model-35 | 34627241 | mRNA | Zhang | ENSG00000085382 | 0.2984 |
| Model-35 | 34627241 | mRNA | Zhang | ENSG00000158270 | 0.1783 |
| Model-35 | 34627241 | mRNA | Zhang | ENSG00000165682 | -0.2154 |
| Model-36 | 35549979 | mRNA | Zeng | ENSG00000100604 | 0.1011 |
| Model-36 | 35549979 | mRNA | Zeng | ENSG00000129521 | 0.1163 |
| Model-36 | 35549979 | mRNA | Zeng | ENSG00000116785 | -0.0940 |
| Model-37 | 34866477 | mRNA | Li | ENSG00000106462 | 0.3934 |
| Model-37 | 34866477 | mRNA | Li | ENSG00000162409 | 0.1436 |
| Model-37 | 34866477 | mRNA | Li | ENSG00000118785 | 0.0832 |
| Model-38 | 33828988 | mRNA | Lin | ENSG00000139514 | 0.1180 |
| Model-38 | 33828988 | mRNA | Lin | ENSG00000104312 | 0.1140 |
| Model-38 | 33828988 | mRNA | Lin | ENSG00000167207 | 0.1130 |
| Model-38 | 33828988 | mRNA | Lin | ENSG00000170425 | 0.0220 |
| Model-38 | 33828988 | mRNA | Lin | ENSG00000112818 | 0.0580 |
| Model-38 | 33828988 | mRNA | Lin | ENSG00000161638 | 0.0510 |
| Model-38 | 33828988 | mRNA | Lin | ENSG00000135124 | 0.0160 |
| Model-38 | 33828988 | mRNA | Lin | ENSG00000106366 | 0.0180 |
| Model-39 | 34221187 | mRNA | Jiang | ENSG00000198492 | 0.0700 |
| Model-39 | 34221187 | mRNA | Jiang | ENSG00000149658 | 0.0200 |
| Model-39 | 34221187 | mRNA | Jiang | ENSG00000165819 | 0.1100 |
| Model-39 | 34221187 | mRNA | Jiang | ENSG00000164944 | 0.0400 |
| Model-39 | 34221187 | mRNA | Jiang | ENSG00000123200 | -0.1000 |
| Model-40 | 32518728 | mRNA | Zhu | ENSG00000160211 | 0.0016 |
| Model-40 | 32518728 | mRNA | Zhu | ENSG00000153395 | 0.0031 |
| Model-40 | 32518728 | mRNA | Zhu | ENSG00000065833 | 0.0063 |
| Model-40 | 32518728 | mRNA | Zhu | ENSG00000198056 | 0.0026 |
| Model-40 | 32518728 | mRNA | Zhu | ENSG00000171848 | 0.0098 |
| Model-40 | 32518728 | mRNA | Zhu | ENSG00000198431 | 0.0078 |
| Model-40 | 32518728 | mRNA | Zhu | ENSG00000143179 | 0.0573 |
| Model-40 | 32518728 | mRNA | Zhu | ENSG00000084774 | 0.0616 |
| Model-40 | 32518728 | mRNA | Zhu | ENSG00000168393 | 0.0085 |
| Model-40 | 32518728 | mRNA | Zhu | ENSG00000054179 | 0.0369 |
| Model-41 | 29678742 | mRNA | Wang | ENSG00000185739 | -0.2700 |
| Model-41 | 29678742 | mRNA | Wang | ENSG00000105948 | 0.1100 |
| Model-41 | 29678742 | mRNA | Wang | ENSG00000165934 | 0.3500 |
| Model-41 | 29678742 | mRNA | Wang | ENSG00000165632 | 0.3700 |
| Model-41 | 29678742 | mRNA | Wang | ENSG00000166455 | -0.2000 |
| Model-41 | 29678742 | mRNA | Wang | ENSG00000126545 | 0.4600 |
| Model-42 | 35356430 | mRNA | Zhou | ENSG00000085998 | 0.0020 |
| Model-42 | 35356430 | mRNA | Zhou | ENSG00000000419 | 0.2310 |
| Model-42 | 35356430 | mRNA | Zhou | ENSG00000158850 | 0.2220 |
| Model-42 | 35356430 | mRNA | Zhou | ENSG00000117411 | 0.1220 |
| Model-42 | 35356430 | mRNA | Zhou | ENSG00000135454 | 0.2120 |
| Model-42 | 35356430 | mRNA | Zhou | ENSG00000149541 | 0.3040 |
| Model-43 | 35693077 | mRNA | Wang | ENSG00000050748 | -0.1670 |
| Model-43 | 35693077 | mRNA | Wang | ENSG00000115902 | -0.0860 |
| Model-43 | 35693077 | mRNA | Wang | ENSG00000100889 | -0.1670 |
| Model-43 | 35693077 | mRNA | Wang | ENSG00000123983 | 0.2030 |
| Model-43 | 35693077 | mRNA | Wang | ENSG00000117632 | 0.2010 |
| Model-43 | 35693077 | mRNA | Wang | ENSG00000129596 | -0.0030 |
| Model-43 | 35693077 | mRNA | Wang | ENSG00000081041 | 0.1090 |
| Model-44 | 32887635 | mRNA | Zhao | ENSG00000148459 | 0.3370 |
| Model-44 | 32887635 | mRNA | Zhao | ENSG00000151012 | 0.3830 |
| Model-44 | 32887635 | mRNA | Zhao | ENSG00000134690 | 0.3560 |
| Model-45 | 35573678 | mRNA | Zhang | ENSG00000089685 | 0.1451 |
| Model-45 | 35573678 | mRNA | Zhang | ENSG00000161011 | 0.1964 |
| Model-45 | 35573678 | mRNA | Zhang | ENSG00000116478 | 0.3711 |
| Model-45 | 35573678 | mRNA | Zhang | ENSG00000106615 | 0.3771 |
| Model-45 | 35573678 | mRNA | Zhang | ENSG00000138363 | 0.3467 |
| Model-45 | 35573678 | mRNA | Zhang | ENSG00000160211 | 0.1620 |
| Model-45 | 35573678 | mRNA | Zhang | ENSG00000278540 | 0.4035 |
| Model-45 | 35573678 | mRNA | Zhang | ENSG00000105281 | 0.2056 |
| Model-45 | 35573678 | mRNA | Zhang | ENSG00000030110 | 0.2847 |
| Model-45 | 35573678 | mRNA | Zhang | ENSG00000105928 | 0.4482 |
| Model-46 | 32813933 | mRNA | Hu | ENSG00000164398 | -0.4665 |
| Model-46 | 32813933 | mRNA | Hu | ENSG00000153395 | 0.0321 |
| Model-46 | 32813933 | mRNA | Hu | ENSG00000170890 | 0.0541 |
| Model-46 | 32813933 | mRNA | Hu | ENSG00000213398 | -0.0219 |
| Model-46 | 32813933 | mRNA | Hu | ENSG00000136699 | 0.1444 |
| Model-47 | 33824863 | mRNA | Weng | ENSG00000049860 | 0.0065 |
| Model-47 | 33824863 | mRNA | Weng | ENSG00000138109 | -0.0006 |
| Model-47 | 33824863 | mRNA | Weng | ENSG00000138363 | 0.0229 |
| Model-47 | 33824863 | mRNA | Weng | ENSG00000160211 | 0.0016 |
| Model-47 | 33824863 | mRNA | Weng | ENSG00000153395 | 0.0082 |
| Model-47 | 33824863 | mRNA | Weng | ENSG00000167325 | 0.0291 |
| Model-47 | 33824863 | mRNA | Weng | ENSG00000171848 | 0.0136 |
| Model-47 | 33824863 | mRNA | Weng | ENSG00000198431 | 0.0122 |
| Model-47 | 33824863 | mRNA | Weng | ENSG00000065833 | 0.0065 |
| Model-47 | 33824863 | mRNA | Weng | ENSG00000248144 | -0.0010 |

1. Li YT, Zeng XZ. Identification of Pyroptosis Gene Signature Related Molecular Pattern, Clinical Implication, and Tumor Immunity in Hepatocellular Carcinoma`. Combinatorial chemistry & high throughput screening 2023; 26(7): 1324-36.

2. Xu K, Liu Y, Luo H, Wang T. Efferocytosis signatures as prognostic markers for revealing immune landscape and predicting immunotherapy response in hepatocellular carcinoma. Frontiers in pharmacology 2023; 14: 1218244.

3. Dai T, Li J, Liang RB, Yu H, Lu X, Wang G. Identification and Experimental Validation of the Prognostic Significance and Immunological Correlation of Glycosylation-Related Signature and ST6GALNAC4 in Hepatocellular Carcinoma. Journal of hepatocellular carcinoma 2023; 10: 531-51.

4. Chen X, Yang M, Wang L, et al. Identification and in vitro and in vivo validation of the key role of GSDME in pyroptosis-related genes signature in hepatocellular carcinoma. BMC cancer 2023; 23(1): 411.

5. Xiong X, Song Q, Jing M, Yan W. Identification of PANoptosis-Based Prognostic Signature for Predicting Efficacy of Immunotherapy and Chemotherapy in Hepatocellular Carcinoma. Genetics research 2023; 2023: 6879022.

6. Sun L, Liu Z, Wu Z, et al. Molecular subtype identification and signature construction based on Golgi apparatus-related genes for better prediction prognosis and immunotherapy response in hepatocellular carcinoma. Frontiers in immunology 2023; 14: 1113455.

7. Zheng C, Peng Y, Wang H, Wang Y, Liu L, Zhao Q. Identification and Validation of Ferroptosis-Related Subtypes and a Predictive Signature in Hepatocellular Carcinoma. Pharmacogenomics and personalized medicine 2023; 16: 39-58.

8. Sun L, Wu Z, Dong C, et al. Signature construction and molecular subtype identification based on immune-related genes for better prediction of prognosis in hepatocellular carcinoma. BMC medical genomics 2023; 16(1): 130.

9. Song H, Ge Y, Xu J, et al. Identification and validation of novel signature associated with hepatocellular carcinoma prognosis using Single-cell and WGCNA analysis. International journal of medical sciences 2023; 20(7): 870-87.

10. Long S, Chen Y, Wang Y, Yao Y, Xiao S, Fu K. Identification of Ferroptosis-related molecular model and immune subtypes of hepatocellular carcinoma for individual therapy. Cancer Med 2023; 12(2): 2134-47.

11. Zhou Y, Lei D, Hu G, Luo F. A Cell Cycle-Related 13-mRNA Signature to Predict Prognosis in Hepatocellular Carcinoma. Frontiers in oncology 2022; 12: 760190.

12. Li M, Gao X, Wang X. Identification of tumor mutation burden-associated molecular and clinical features in cancer by analyzing multi-omics data. Frontiers in immunology 2023; 14: 1090838.

13. Chi H, Zhao S, Yang J, et al. T-cell exhaustion signatures characterize the immune landscape and predict HCC prognosis via integrating single-cell RNA-seq and bulk RNA-sequencing. Frontiers in immunology 2023; 14: 1137025.

14. Chen Y, Tang L, Huang W, et al. Identification of a prognostic cuproptosis-related signature in hepatocellular carcinoma. Biology direct 2023; 18(1): 4.

15. Du B, Wang F, Jarad B, Wang Z, Zhang Y. A novel signature based on microvascular invasion predicts the recurrence of HCC. Journal of translational medicine 2020; 18(1): 272.

16. Zhao Y, Zhang J, Wang S, Jiang Q, Xu K. Identification and Validation of a Nine-Gene Amino Acid Metabolism-Related Risk Signature in HCC. Frontiers in cell and developmental biology 2021; 9: 731790.

17. Zhang S, Li X, Zhang X, Zhang S, Tang C, Kuang W. The Pyroptosis-Related Gene Signature Predicts the Prognosis of Hepatocellular Carcinoma. Frontiers in molecular biosciences 2021; 8: 781427.

18. Hu Y, Liu J, Yu J, et al. Identification and validation a costimulatory molecule gene signature to predict the prognosis and immunotherapy response for hepatocellular carcinoma. Cancer cell international 2022; 22(1): 97.

19. Zhu G, Xia H, Tang Q, Bi F. An epithelial-mesenchymal transition-related 5-gene signature predicting the prognosis of hepatocellular carcinoma patients. Cancer cell international 2021; 21(1): 166.

20. Peng Y, Liu C, Li M, et al. Identification of a prognostic and therapeutic immune signature associated with hepatocellular carcinoma. Cancer cell international 2021; 21(1): 98.

21. Yuan C, Yuan M, Chen M, et al. Prognostic Implication of a Novel Metabolism-Related Gene Signature in Hepatocellular Carcinoma. Frontiers in oncology 2021; 11: 666199.

22. Xie H, Liu S, Zhang Z, Chen P, Tao Y. A novel seven-gene signature as Prognostic Biomarker in Hepatocellular Carcinoma. Journal of Cancer 2020; 11(19): 5768-81.

23. Hui Y, Leng J, Jin D, et al. A Cell Cycle Progression-Derived Gene Signature to Predict Prognosis and Therapeutic Response in Hepatocellular Carcinoma. Disease markers 2021; 2021: 1986159.

24. Liu Z, Jiao D, Liu L, et al. Development and validation of a robust immune-related risk signature for hepatocellular carcinoma. Medicine 2021; 100(10): e24683.

25. Xu W, Chen Z, Liu G, et al. Identification of a Potential PPAR-Related Multigene Signature Predicting Prognosis of Patients with Hepatocellular Carcinoma. PPAR research 2021; 2021: 6642939.

26. Jiang HY, Ning G, Wang YS, Lv WB. Ahypoxia-related signature enhances the prediction of the prognosis in hepatocellular carcinoma patients and correlates with sorafenib treatment response. American journal of translational research 2020; 12(12): 7762-81.

27. Su L, Zhang G, Kong X. A Novel Five-Gene Signature for Prognosis Prediction in Hepatocellular Carcinoma. Frontiers in oncology 2021; 11: 642563.

28. Huang C, Zhang C, Sheng J, et al. Identification and Validation of a Tumor Microenvironment-Related Gene Signature in Hepatocellular Carcinoma Prognosis. Frontiers in genetics 2021; 12: 717319.

29. Zhao E, Chen S, Dang Y. Development and External Validation of a Novel Immune Checkpoint-Related Gene Signature for Prediction of Overall Survival in Hepatocellular Carcinoma. Frontiers in molecular biosciences 2020; 7: 620765.

30. He J, Ran J, Li J, Chen D. Construction and validation of a pyroptosis-related gene signature in hepatocellular carcinoma based on RNA sequencing. Translational cancer research 2022; 11(6): 1510-22.

31. Mo Z, Zhang S, Zhang S. A Novel Signature Based on mTORC1 Pathway in Hepatocellular Carcinoma. Journal of oncology 2020; 2020: 8291036.

32. Chen J, Wang H, Zhou L, Liu Z, Chen H, Tan X. A necroptosis-related gene signature for predicting prognosis, immune landscape, and drug sensitivity in hepatocellular carcinoma. Cancer Med 2022; 11(24): 5079-96.

33. Hong L, Zhou Y, Xie X, et al. A stemness-based eleven-gene signature correlates with the clinical outcome of hepatocellular carcinoma. BMC cancer 2021; 21(1): 716.

34. Zhang BH, Yang J, Jiang L, et al. Development and validation of a 14-gene signature for prognosis prediction in hepatocellular carcinoma. Genomics 2020; 112(4): 2763-71.

35. Zhang G, Su L, Lv X, Yang Q. A novel tumor doubling time-related immune gene signature for prognosis prediction in hepatocellular carcinoma. Cancer cell international 2021; 21(1): 522.

36. Zeng Z, Lei S, Wang J, et al. A novel hypoxia-driven gene signature that can predict the prognosis of hepatocellular carcinoma. Bioengineered 2022; 13(5): 12193-210.

37. Li X, Lin J, Pan Y, Cui P, Xia J. Identification of a Liver Progenitor Cell-Related Genes Signature Predicting Overall Survival for Hepatocellular Carcinoma. Technology in cancer research & treatment 2021; 20: 15330338211041425.

38. Lin Z, Xu Q, Miao D, Yu F. An Inflammatory Response-Related Gene Signature Can Impact the Immune Status and Predict the Prognosis of Hepatocellular Carcinoma. Frontiers in oncology 2021; 11: 644416.

39. Jiang H, Ning G, Wang Y, Lv W. Identification of an m6A-Related Signature as Biomarker for Hepatocellular Carcinoma Prognosis and Correlates with Sorafenib and Anti-PD-1 Immunotherapy Treatment Response. Disease markers 2021; 2021: 5576683.

40. Zhu Z, Li L, Xu J, et al. Comprehensive analysis reveals a metabolic ten-gene signature in hepatocellular carcinoma. PeerJ 2020; 8: e9201.

41. Wang Z, Teng D, Li Y, Hu Z, Liu L, Zheng H. A six-gene-based prognostic signature for hepatocellular carcinoma overall survival prediction. Life sciences 2018; 203: 83-91.

42. Zhou Z, Wang T, Du Y, Deng J, Gao G, Zhang J. Identification of a Novel Glycosyltransferase Prognostic Signature in Hepatocellular Carcinoma Based on LASSO Algorithm. Frontiers in genetics 2022; 13: 823728.

43. Wang H, Yang C, Jiang Y, Hu H, Fang J, Yang F. A novel ferroptosis-related gene signature for clinically predicting recurrence after hepatectomy of hepatocellular carcinoma patients. American journal of cancer research 2022; 12(5): 1995-2011.

44. Zhang B, Tang B, Gao J, Li J, Kong L, Qin L. A hypoxia-related signature for clinically predicting diagnosis, prognosis and immune microenvironment of hepatocellular carcinoma patients. Journal of translational medicine 2020; 18(1): 342.

45. Zhang G, Fan W, Wang H, et al. Non-Apoptotic Programmed Cell Death-Related Gene Signature Correlates With Stemness and Immune Status and Predicts the Responsiveness of Transarterial Chemoembolization in Hepatocellular Carcinoma. Frontiers in cell and developmental biology 2022; 10: 844013.

46. Hu B, Yang XB, Sang XT. Construction of a lipid metabolism-related and immune-associated prognostic signature for hepatocellular carcinoma. Cancer Med 2020; 9(20): 7646-62.

47. Weng J, Zhou C, Zhou Q, et al. Development and Validation of a Metabolic Gene-Based Prognostic Signature for Hepatocellular Carcinoma. Journal of hepatocellular carcinoma 2021; 8: 193-209.
