# Supplementary material for: Mitotic catastrophe heterogeneity: implications for prognosis and immunotherapy in hepatocellular carcinoma
Source: Front Immunol. 2024 Jul 1;15:1409448. doi: 10.3389/fimmu.2024.1409448 (PMC11250588; doi:10.3389/fimmu.2024.1409448)
Supplement: Supplementary Figure 1 — Study design and analytical workflow. [file Image 16.pdf › Image 16.pdf]

Figure S16

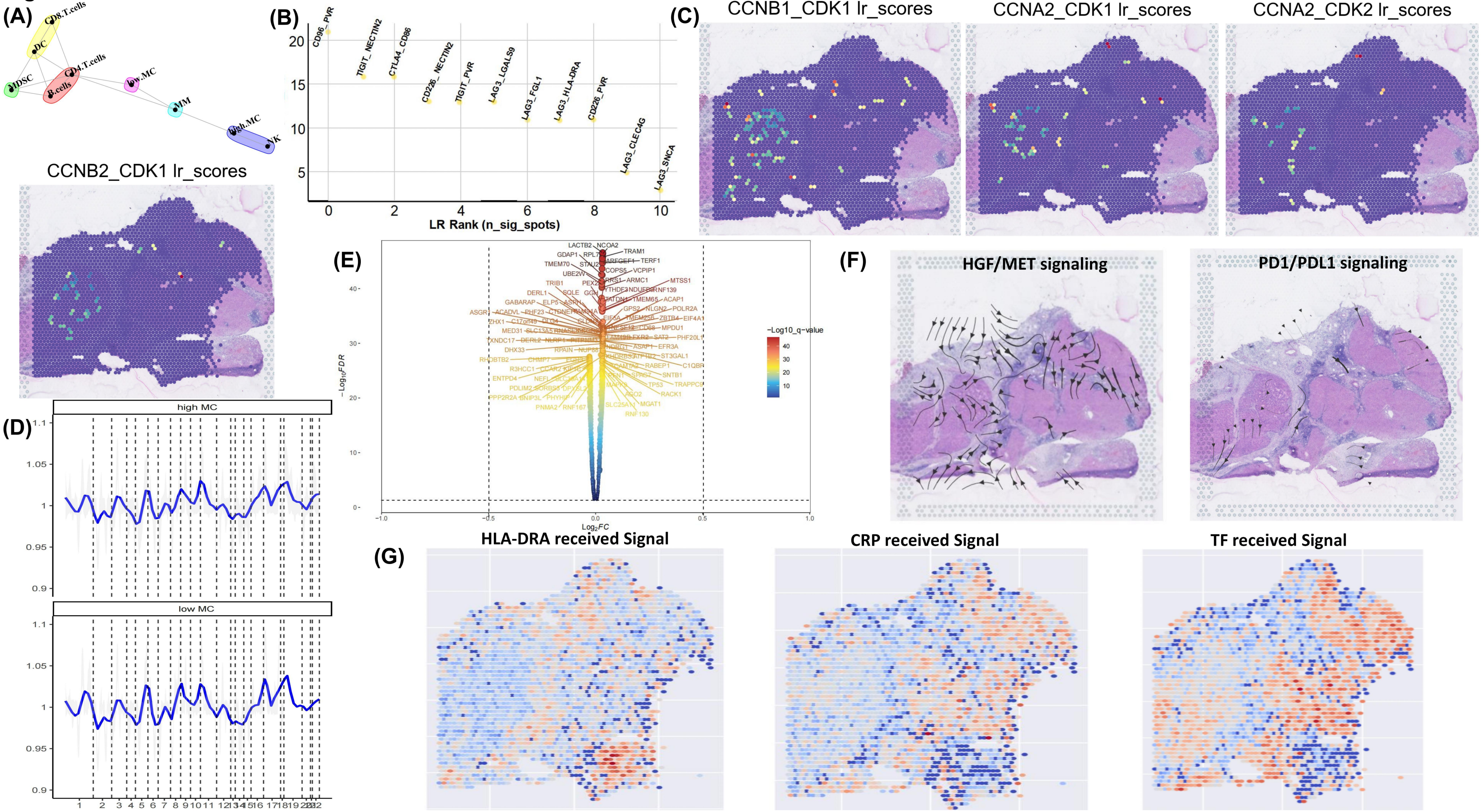

(A)
CD8.T.cells
DC
CD4.T.cells
MDSC
B.cells
low.MC
MM
high.MC
NK
(B)
CD96_PVR
TIGIT_NECTIN2
CTLA4_CD86
CD226_NECTIN2
TIGIT_PVR
LAG3_LGALS9
LAG3_FGL1
LAG3_HLA-DRA
CD226_PVR
LAG3_CLEC4G
LAG3_SNCA
LR Rank (n_sig_spots)
(C)
CCNB1_CDK1 lr_scores
CCNA2_CDK1 lr_scores
CCNA2_CDK2 lr_scores
CCNB2_CDK1 lr_scores
(D)
high MC
low MC
(E)
−Log10_q-value
Log2FC
(F)
HGF/MET signaling
PD1/PDL1 signaling
(G)
HLA-DRA received Signal
CRP received Signal
TF received Signal
